# Effects of behavioural exercise therapy on the effectiveness of multidisciplinary rehabilitation for chronic non-specific low back pain: a randomised controlled trial

**DOI:** 10.1186/s12891-021-04353-y

**Published:** 2021-05-29

**Authors:** Jana Semrau, Christian Hentschke, Stefan Peters, Klaus Pfeifer

**Affiliations:** 1 Department of Sport Science and Sport, Friedrich-Alexander-University Erlangen-Nuremberg, Gebbertstraße 123 b, 91056 Erlangen, Germany; 2 Novartis Pharma GmbH, Roonstraße 25, 90429 Nuremberg, Germany; 3 Deutscher Verband für Gesundheitssport und Sporttherapie (DVGS) e.V, Vogelsanger Weg 48, 50354 Hürth-Efferen, Germany

**Keywords:** Behavioural exercise therapy, Multidisciplinary biopsychosocial rehabilitation, Chronic low back pain, Biopsychosocial framework

## Abstract

**Background:**

The long-term effects of behavioural medical rehabilitation (BMR), as a type of multidisciplinary rehabilitation, in the treatment of chronic non-specific low back pain (CLBP) have been shown. However, the specific effects of behavioural exercise therapy (BET) compared to standard exercise therapy (SET) within BMR are not well understood. The aim of the study was to assess the effectiveness of BMR + BET compared to BMR + SET in individuals with CLBP in a two-armed, pre-registered, multicentre, parallel, randomised controlled trial (RCT).

**Methods:**

A total of 351 adults with CLBP in two rehabilitation centres were online randomised based on an ‘urn randomisation’ algorithm to either BMR + SET (*n* = 175) or BMR + BET (*n* = 176). Participants in both study groups were non-blinded and received BMR, consisting of an multidisciplinary admission, a psychosocial assessment, multidisciplinary case management, psychological treatment, health education and social counselling. The intervention group (BMR + BET) received a manualised, biopsychosocial BET within BMR. The aim of BET was to develop self-management strategies in coping with CLBP. The control group (BMR + SET) received biomedical SET within BMR with the aim to improve mainly physical fitness. Therapists in both study groups were not blinded. The BMR lasted on average 27 days, and both exercise programmes had a mean duration of 26 h. The primary outcome was functional ability at 12 months. Secondary outcomes were e.g. pain, avoidance-endurance, pain management and physical activity. The analysis was by intention-to-treat, blinded to the study group, and used a linear mixed model.

**Results:**

There were no between-group differences observed in function at the end of the BMR (mean difference, 0.08; 95% CI − 2.82 to 2.99; *p* = 0.955), at 6 months (mean difference, − 1.80; 95% CI; − 5.57 to 1.97; *p* = 0.349) and at 12 months (mean difference, − 1.33; 95% CI − 5.57 to 2.92; *p* = 0.540). Both study groups improved in the primary outcome and most secondary outcomes at 12 months with small to medium effect sizes.

**Conclusion:**

BMR + BET was not more effective in improving function and other secondary outcomes in individuals with CLBP compared to BMR + SET.

**Trial registration:**

Current controlled trials NCT01666639, 16/08/2012.

**Supplementary Information:**

The online version contains supplementary material available at 10.1186/s12891-021-04353-y.

## Background

Chronic low back pain (CLBP) is one of the leading causes of years lived with disability worldwide [1]. In Germany, musculoskeletal and connective tissue diseases are the most common conditions of adults who receive a traditional multidisciplinary rehabilitation [2]. CLBP is the most prevalent health condition in this category. Traditional multidisciplinary rehabilitation is the main type of rehabilitation in Germany and aims to improve function and work ability. It mostly takes place in an inpatient setting and lasts about 3 weeks.

According to current evidence, multidisciplinary biopsychosocial rehabilitation should be the treatment of choice for individuals with disabling CLBP who have not responded to other treatment options [3–5]. With behavioural medical rehabilitation (BMR) [6, 7], a type of multidisciplinary rehabilitation with an explicitly biopsychosocial approach is being used in Germany for individuals with disabling CLBP. The overall mechanism of action of a BMR is based on the biopsychosocial model for low back pain as described by Waddell et al. [8]. Accordingly, CLBP and its associated disability are understood to result from complex interactions between many different physical, psychological and social factors who overlap and interact with each other [5, 9]. Within this biopsychosocial framework, BMR follows a cognitive-behavioural approach [7, 10–12] with the aim to identify and alter harmful beliefs, emotions, and behaviour regarding pain and function.

BMR has been proven to be superior in comparison to traditional multidisciplinary rehabilitation in individuals with musculoskeletal disorders regarding depression in the short term and regarding selected strategies for coping with pain in the long term [6]. It has also been shown to improve subjective health status [13]. A meta-analysis [5], whilst including BMR, detected long-term improvements with small to medium effect sizes of multidisciplinary biopsychosocial rehabilitation regarding the outcomes of pain and disability compared to usual care or interventions mainly targeting physical factors.

BMR includes standard exercise therapy (SET), which is mainly based on a biomedical approach with methods aiming at an improvement in physical fitness [6]. SET usually accounts for about 68% of all interventions in traditional multidisciplinary rehabilitation in Germany [14]. The potential underlying mechanisms of action for SET [15–17] to reduce disabling CLBP are improvements in several domains: strength and endurance of back muscles, trunk flexibility, bone strength, blood supply to spinal muscles, joints, and intervertebral discs, body composition, and cardiorespiratory fitness. Those improvements could supposedly contribute to a healing process in body functions and structures, which in turn could lead to reduced pain and improved function [16, 17].

Although SET is embedded in a BMR [6, 7, 12], it does not contain contents or methods for targeting psychosocial factors [18–22] that have been shown to be related to the development of disabling CLBP. This could be an obstacle to recovery and improved function in individuals with CLBP [23–27].

By contrast, exercise therapy based on frameworks of behaviour change is lacking within German rehabilitation [28]. BET, as conceptualised by [29], goes beyond a mere biomedical approach by addressing personal context factors such as the above-mentioned psychosocial factors in individuals with CLBP [18, 26] and systematically integrates behaviour change techniques [30] to promote physical activity. The other aims of BET are to improve health-related fitness and to improve the self-management of CLBP [12]. Such a theory-based intervention seems advantageous in fostering long-term adherence to a physically active lifestyle due to evidence across medical conditions [31] and in musculoskeletal conditions in particular [32].

However, there is a need for a better understanding of the mechanisms of action of multidisciplinary biopsychosocial rehabilitation [5, 18, 33] and the specific effects of BET on the long-term effects of such programmes as this is the basis for improved treatment efficacy and decisions regarding who might benefit the most from this treatment option [18].

Therefore, the aim of the study was to assess the specific effects of BMR with BET compared to BMR with SET in individuals with CLBP in a two-armed, pre-registered, multicentre, parallel, randomised controlled trial (RCT). It is hypothesised that a behavioural approach to exercise therapy (BET) [12], which addresses both relevant psychosocial factors of pain chronification [34–36] and determinants of physical activity behaviour change [29, 30, 32], improves the long-term effectiveness of BMR compared to BMR with SET.

## Methods

### Study design and procedure

This study was conducted according to the recommendations of the World Medical Association (Declaration of Helsinki: [37]). Ethical approval had been granted by the independent Research Ethics Committee of the Medical Faculty of Friedrich-Alexander-University of Erlangen-Nuremberg (Re.-No. 4510). The study was registered prospectively (ClinicalTrials.gov: NCT01666639) and the study protocol, in line with the Consort Statement [38], was published in this journal [12].

It was a multicentre, prospective, two-arm parallel randomised controlled trial with four measurement time points conducted in two rehabilitation centres in Germany.

In Germany, the need for rehabilitation is determined by general practitioners or company physicians. With their diagnostic findings, a patient applies for authorisation of rehabilitation at the German Pension Insurance Association, which examines internally whether the application meets the requirements for a BMR. In case of a positive result, the association informs the patient with regard to the rehabilitation centre in which the BMR will take place. The exact date of admittance is determined individually in the respective centre.

For the study, recruitment was conducted consecutively between January 2012 and March 2013, after 6 months between July 2012 and September 2013, and the one-year follow-up was finished in March 2014. After the submission of the signed informed consent of the study participants, enrolment took place based on the assessment of the inclusion and exclusion criteria (see Table 1) by the physicians within the inpatient rehabilitation centres.

**Table 1:** Inclusion and Exclusion Criteria

Inclusion criteria	Exclusion criteria
• M51.2-M51.9 (other intervertebral disc disorders) • M53.8, M53.9 (other specified/unspecified dorsopathies) • M54.4-M54.9 (lumbago with sciatica, low back pain, pain in thoracic spine, other/unspecified dorsalgia) • F45.4 (persistent somatoform pain disorder) • F45.41 (chronic pain disorder with somatic and psychological factors) • F54 (psychological and behavioural factors associated with disorders or diseases classified elsewhere)	• specific underlying diagnosis of the back pain (e.g. radicular symptoms, myelopathy) • considerably reduced health status (comorbidities) • considerable reduction of sight and hearing • severe psychiatric condition as secondary diagnosis • age below 18 or over 65 • lack of ability to speak German • ongoing application for retirement

The recruitment procedure was adjusted to each of the centre’s internal structures and processes regarding the invitation of participants. In the rehabilitation centre “Paracelsus-Klinik an der Gande”, potential study participants were identified by a screening of the record of participants by the head physician prior to the invitation to the BMR. Afterwards, eligible participants obtained a written participant information and a consent form about the study and were asked to participate. After returning the signed consent form within 7 days, the participants were randomised online. The invitation of the randomised participants took place weekly and in groups according to the group assignment in the respective week of arrival. At the beginning of the BMR, an information meeting was held by the head physician for all eligible participants, explaining the study in detail.

In the rehabilitation centre “Klinik Weser”, potential study participants were identified at the beginning of the BMR in medical admission interviews by physicians and obtained a written consent form. In an information meeting, all eligible participants were informed about the study comprehensively by the head physician. On the second day, after returning the signed consent form, the online-based randomisation took place. Due to spatial and personnel-wise requirements, one control and one intervention group started simultaneously every fourteen days. Therefore, the recruitment of eligible participants took place in a biweekly rhythm.

Primary and secondary outcomes were measured with standardised questionnaires directly at the start of the BMR (t1), after 4 weeks at the end of it (t2) as well as six (t3) and 12 months (t4) after completion of the BMR. The follow-up questionnaires were sent by post by the project team of the University six and 12 months after the BMR. Postal reminders were sent after 3 weeks in cases where the questionnaire had not been returned.

### Participants

Participants with CLBP were eligible for the study. CLBP was defined as persisting pain for at least 3 months, localised below the costal margin and above the inferior gluteal folds, without referred leg pain and that is not caused by a known specific pathology [39]. Inclusion and exclusion criteria were based on ICD-10 (International Classification of Diseases) [40]).

Both participating inpatient rehabilitation centres are in Lower Saxony, a federal state in northwest Germany. The main medical conditions being treated in the centres are musculoskeletal disorders, with both offering inpatient and outpatient programmes. BMR is provided as an inpatient programme in both rehabilitation centres. The rehabilitation centre “Klinik Weser” has 251 beds and the other, “Paracelsus-Klinik an der Gande”, has 120 beds. The latter is privately owned by the Paracelsus concern (https://bit.ly/3u7cvMJ), but individuals with musculoskeletal disorders are mainly assigned through the German Pension Insurance Association. “Klinik Weser” (https://bit.ly/2OvQojm) is one of 22 rehabilitation centres directly owned by the Association. Both rehabilitation centres were involved in numerous research activities during the last years.

## Intervention

Both participating centres had, based on a common conceptual framework [6, 7], a conceptually similar BMR for participants with musculoskeletal disorders and concurrent substantial psychological or social components of function. The BMR considers the biopsychosocial mechanisms relevant for the chronification of musculoskeletal disorders [7, 8]. Empowerment and self-management are encouraged throughout the intervention. There are no single underlying mechanisms established which might explain the effectiveness of a complex BMR [5, 41]. Therefore, the biopsychosocial framework seems to be the best available framework to describe the complex interplay of biological, psychological, and social factors, which are addressed within the BMR. All treatment components within the BMR are guided by treatment principles based on a cognitive-behavioural approach [12, 42]. However, the psychological treatment specifically addresses beliefs, emotions and behaviour as described in the fear-avoidance [43, 44] or avoidance-endurance model [45]. The psychological therapists further introduce the biopsychosocial model, discuss the role of thoughts and emotions, pain acceptance, sleep hygiene, the relationship between pain and social competence or the development of goals to self-manage pain in everyday life. Further information about content and methods of the psychological treatment within BMR have been described in the study protocol [12]. The SET within a BMR follows a biomedical approach and specifically addresses physical factors (e.g. strength/endurance of back muscles, trunk flexibility, bone strength, blood supply, body composition, cardiorespiratory fitness) [17].

In routine care, the BMR lasts for 27 days on average. Compared to traditional multidisciplinary rehabilitation, the BMR has the following specifically defined criteria [7]: 1) multidisciplinary admission, 2) standardised psychosocial assessment, 3) reconciled multidisciplinary case management, 4) case reviews on a regular basis, 5) supervision, 6) closed groups (groups remain together in all treatments during their whole stay at the rehabilitation centre), 7) the possibility of an individually tailored therapy schedule and 8) therapist consistency (a therapist accompanies a group throughout the whole treatment). The multidisciplinary core of the BMR relies on psychological treatments, which are provided by a psychologist or psychotherapist and SET, which is provided by physical therapists. Other interventions are education about health and health behaviour as well as social counselling. The total extent of therapy during the inpatient stay is 65 h on average, including approximately 26 h of exercise therapy. The multidisciplinary team of an BMR in both rehabilitation centres consists of the physicians, psychologists or psychotherapist, physical therapists, occupational therapists, nutritionists, nurses, and social workers [7].

In each rehabilitation centre, the same multidisciplinary team conducted the BMR in both study groups. But, SET and BET within BMR were provided by different physical therapists as described below.

### Control group (BMR + SET)

The SET within the BMR consisted of a closed group (10 to 12 participants) with eight to 13 sessions and a session duration of 30 to 45 min each. Additionally, participants received an assessment of physical fitness, back school (mainly education about CLBP), general resistance training, water-based therapy, walking/Nordic walking and stationary cycling. The primary aim of the SET within the BMR was to improve physical fitness. The total extent of SET was approximately 26 h. The SET in both rehabilitation centres was conducted by experienced physical therapists. These physical therapists provided only SET and were not involved in BET. Further details are available in the study protocol [12].

### Intervention group (BMR + BET)

The BET within the BMR was also conducted in a closed group with six to 12 participants. BET aimed at 1) the development of active self-management strategies when coping with CLBP, 2) an introduction to physical activity and the long-term maintenance of it and 3) the improvement of health-related fitness. To address these objectives, appropriate exercise elements were systematically combined with health education and behavioural techniques. The main difference between BET and SET is the combination of the three mentioned aims based on a biopsychosocial framework within a coherent closed group format in BET. Exercises to improve physical or health-related fitness were similar in SET and BET.

The promotion of self-management in BET included education and group discussions about different coping strategies in relation to physical activity, the interplay of thoughts, mood and posture and how to deal with recurring episodes of CLBP. Further details are described in Additional file 1, in the study protocol [12] as well as in a detailed manual with media and materials (409 pages, available in German under https://bit.ly/3u69mwz).

The BET consisted of 15 sessions, with a duration of 60 min each. Additionally, participants received two introductory sessions for strength training, two for aerobic exercise (walking, nordic walking and stationary cycling) and two for water-based training during the first week. Moreover, three modules of action planning and barrier management were distributed over 3 weeks. The total extent of the BET was approximately 26 h. The detailed manual was developed to support the therapists in the implementation of the intervention. The 15 sessions and the related modules were guided by trained physical therapists in both rehabilitation centres. In one centre, eight therapists were trained in BET, and two in the other centre. These physical therapists remained the same in both centres during the study period and did not provide SET. Further details are available in the study protocol [12].

### Treatment integrity

The therapists who performed the BET in the intervention group (IG) received an intensive standardised training on objectives, content, structure, components, and methods of the programme based on the manualised intervention concept (see https://bit.ly/3u69mwz). Both workshops were delivered by the same trainers and consisted of 31 lessons of 45 min each. Pilot groups with the BET programme were performed during the implementation phase of the study. To minimise the confounding of the randomisation, trained BET therapists signed a written contract concerning discretion about objectives, content, structure, methods, media and materials used within the BET. In cases of vacation or sick leave, trained replacement therapists were available in both rehabilitation centres. Two announced visitations during the intervention phase were performed to assure treatment adherence [12].

### Outcomes and measurements

The level of function, used as the primary outcome, was measured by the Hannover Functional Ability Questionnaire (HFAQ, 1–100 [46]). The chosen secondary outcome measures were pain (Numeric Rating Scale, NRS, 0–100); depression (Patient Health Questionnaire, PHQ-D, 0–27 [47]); anxiety (General Anxiety Disorder 7-item Scale, GAD-7, 0–21 [48]); health-related quality of life (Short-Form-12, SF-12, 0–100 [49]); stress (Perceived Stress Scale, PSS, 0–40 [50]); physical activity (Freiburg Questionnaire on Physical Activity, FFkA, hours/week [51]); and pain-related emotions, cognitions and behaviour (Pain Management Questionnaire, FESV, 4–24 [52]), Tampa Scale for Kinesiophobia, TSK, 0–15 somatic focus, 0–24 fear-avoidance [44], Avoidance-Endurance Questionnaire, AEQ, 0–6 [45]) as well as emotional, motivational and volitional determinants of physical activity (cognitive attitudinal component, emotional attitudinal component, intention, self-efficacy, risk perception, outcome expectancies, and outcome experiences) [30, 53–58]. Ranges for these measurements are provided in Table 2. Further information is available in the study protocol [12].

**Table 2:** Baseline characteristics

Outcome (unit)	N CG/IG	Control group (CG)	Intervention group (IG)
Age (Year), Mean (SD)	162/165	51 (7.4)	51.24 (7.4)
Sex, Female %	163/164	81.7	75.76
Married/ Partner %	161/164	78.26	66.25
Employed %	161/163	90.68	83.54
Sick Leave due to Back Pain Last six months, Mean (SD)	163/163	17.09 (47.55)	19.67 (49.22)
Severely Handicapped ID % *	163/164	8	10
Functional Ability (HFAQ) (0–100)	163/164	62.28 (20.50)	63.33 (19.95)
Pain Intensity (NRS) (0–100)	159/162	59.99 (17.44)	59.59 (16.06)
Physical Composite Scale (SF-12) (0–100)	152/152	35.46 (9.03)	36.23 (8.39)
Mental Composite Scale (SF-12) (0–100)	152/152	40.32 (12.01)	39.97 (11.92)
Depression (PHQ-D) (0–27)	160/157	9.65 (4.66)	10.01 (5.16)
Anxiety (Gad-7) (0–21)	159/163	8.64 (4.83)	9.29 (5.02)
Stress (PSS) (0–40)	160/162	7.78 (3.20)	7.83 (3.39)
Catastrophising (AEQ) (0–6)	161/164	0.77 (1.11)	0.92 (1.15)
Help−/Hopelessness (AEQ) (0–6)	161/164	2.45 (1.21)	2.52 (1.22)
Thought Suppression (AEQ) (0–6)	163/163	3.64 (0.90)	3.72 (0.94)
Anxiety/Depression (AEQ) (0–6)	161/164	2.64 (1.16)	2.77 (1.28)
Positive Mood (AEQ) (0–6)	160/164	3.22 (1.24)	3.68 (1.24)
Avoidance of Social Activities (AEQ) (0–6)	162/163	1.86 (1.26)	2.07 (1.33)
Avoidance of Physical Activities (AEQ) (0–6)	163/163	3.31 (1.02)	3.49 (0.98)
Humour/Distraction (AEQ) (0–6)	162/163	3.07 (0.89)	3.01 (1.04)
Pain Persistence Behaviour (AEQ) (0–6)	160/164	3.68 (1.24)	3.62 (1.34)
Action-oriented Coping (FESV) (4–24)	161/ 153	14.80 (4.81)	15.02 (4.75)
Cognitive Restructuring (FESV) (4–24)	161/ 153	13.03 (4.58)	13.47 (4.47)
Subjective Coping Competence (FESV) (4–24)	161/ 153	15.45 (4.63)	15.21 (4.41)
Mental Distraction (FESV) (4–24)	161/ 153	10.58 (4.67)	10.39 (4.94)
Counter Activities (FESV) (4–24)	161/ 153	13.21 (4.37)	12.39 (4.44)
Relaxation (FESV) (4–24)	161/ 153	10.77 (4.92)	11.17 (4.66)
Fear-Avoidance (TSK) (0–24)	158/158	6.87 (3.26)	7.54 (3.53)
Somatic focus (TSK) (0–15)	159/160	6.78 (3.43)	7.17 (3.45)
Basic Physical Activity (FFkA) (hours/week)	161/163	2.89 (6.65)	2.44 (4.80)
Physical Activity during Leisure Time (FFkA) (hours/week)	161/164	3.22 (4.39)	2.72 (3.27)
Sport Activity (FFkA) (hours/week)	162/164	1.69 (2.74)	1.58 (2.64)
Total Physical Activity (FFkA) (hours/week)	161/163	7.82 (10.63)	6.73 (7.47)
Cognitive Attitudinal Component (0–8)	161/164	5.32 (0.77)	5.28 (0.80)
Emotional Attitudinal Component (0–8)	160/164	4.75 (0.81)	4.70 (0.88)
Intention (1–4)	162/164	3.30 (0.54)	3.29 (0.50)
Self-efficacy (1–4)	162/164	3.20 (0.55)	3.19 (0.60)
Action Planning (1–4)	162/162	2.88 (0.74)	2.85 (0.81)
Risk Perception (1–5)	161/162	3.23 (0.73)	3.28 (0.76)
Outcome Expectancies (1–4)	163/164	2.75 (0.31)	2.74 (0.28)
Outcome Experiences (1–4)	160/164	2.73 (0.65)	2.74 (0.80)
Action Control (1–4)	160/162	2.49 (0.67)	2.54 (0.78)

*N* number of participants, *SD* standard deviation: * = *p* < 0.05, *ID* identity card, *HFAQ* Hannover Functional Ability Questionnaire, *NRS* Numeric Rating Scale, *PHQ-D* Patient Health Questionnaire, *GAD-7* General Anxiety Disorder 7-item Scale, *PSS* Perceived Stress Scale, *AEQ* Avoidance-Endurance Questionnaire, *FESV* Pain Management Questionnaire, *TSK* Tampa Scale for Kinesiophobia, *FFkA* Freiburg Questionnaire on Physical Activity, *SF-12* Short-Form-12

Physical functioning (e.g. HFQA), health-related quality of life (e.g. SF-12) and pain intensity (e.g. NRS) are recommended core outcome measures for clinical trials in nonspecific low back pain [59, 60]. The NIH task force [60] further recommends depression (e.g. PHQ-D) and catastrophizing (e.g. AEQ) as outcomes in CLBP. Pain management (FESV), anxiety (GAD-7), and stress (PSS) are important outcome domains to assess the effects of the cognitive-behavioural approach used in BMR and BET and were also used in previous studies [6, 61]. The Tampa Scale for Kinesiophobia [44, 62] captures fear of movement/(re) injury and is a widely accepted measure to analyse the fear-avoidance related pathway to CLBP and function. The Avoidance-Endurance Questionnaire [45] assesses, in addition to the fear-avoidance-related pathway, a second one with endurance-related responses to chronic pain and allows to distinguish between affective, cognitive and behavioural responses to pain within a biopsychosocial framework. These psychosocial factors are addressed in the cognitive-behavioural approach of BMR and BET but not in SET. All outcome measures are equally important to gain deeper insights regarding the contribution of psychosocial factors within a biopsychosocial framework on disabling CLBP. Physical activity as well as its emotional, motivational, and volitional determinants are addressed by health behaviour change techniques [30–32] with the aim to develop a physically active lifestyle. These techniques were only used in BET. The choice of the primary outcome and the secondary outcome measures reflects the focus of this study on the influences of a behavioural approach to exercise therapy on the effectiveness of BMR.

With exception of physical activity, all outcomes were assessed at all four time points. Physical activity was assessed at start of rehabilitation (t1), after 6 months (t3) and after 12 months (t4). As the physical activity level during the inpatient BMR is not comparable to the situation before and after the BMR, we decided not to measure physical activity at the end of BMR (t2).

### Randomisation

Participants with CLPB were randomised into either a) the usual BMR with SET as the control group (CG) or b) BMR with the BET as the intervention group (IG). The randomisation of eligible participants took place before completion of the t1 questionnaire using an online-based randomisation feature. For that purpose, we implemented a central data base for electronic data recording which was used in each of the two rehabilitation centres. In both of them, an independent employee, who was not involved in the study, used the online-based randomisation to register eligible participants via a web application. After registration, the online-based randomisation automatically carried out the allocation to one of the treatment groups. This system has been developed in a prior study [63] and incorporates the existing national data protection regulations. Clear advantages of this procedure are a prompt randomisation and a concealment of the sequence of allocation. For sequence generation, we used an ‘urn randomisation’ algorithm [64] which has good statistical properties [65] and can be used to stratify according to different criteria. Stratification was performed for ‘rehabilitation centre’ and ‘gender’, with two categories each.

### Blinding

The blinding of the study’s therapists was not possible because they were either trained to perform the BET or were chosen to deliver the existing exercise therapy within the BMR during the study period. Participants were masked regarding the treatment group and were not informed as to whether they were participating in the CG or IG during the study period. A statistician who was not involved in the study process performed the statistical data analysis. Furthermore, the evaluation was blinded to the treatment group.

### Statistical analyses

The level of function associated with CLBP, 12 months after the end of the BMR, was used as the primary outcome [46]. Regarding the short-term effects of both interventions, only small differences between groups and small effect sizes at the end of the BMR (t2) (d = 0,3 or f(V) = 0,15) were expected to be found [5, 25, 26]. Because of the minor long-term effects of the BMR, medium effect sizes (d = 0,5 or f(V) = 0,25) were expected at six and 12 months. Sample power (Software: G-Power 3.0) concerning the primary outcome was approximated for an analysis of covariance at 12 months (t4) with a medium effect size of Cohen’s d = 0,5, an alpha error of 5% and a test power of 80%. This approximation resulted in a sample size of at least 128 participants without missing values. Anticipating a dropout rate of 40%, it was necessary to include 214 participants (*n* = 107 for each study group).

Statistical analyses were performed using R statistics [66]. Baseline differences in demographics as well as the primary and secondary outcomes data were analysed using two-sample t-tests for parametric and the Wilcoxon test for non-parametric distribution as well as the chi-squared test for nominal data.

The chance for dropout was estimated using logistic regression. Therefore, the known binary variable of early study discontinuation was included as depended variable. The selection of predictors followed an explorative approach based on the explained overall variance (Nagelkerkes R2) and the statistical significance of the given predictor. As candidate predictors all baseline variables and also some post baseline measures of several outcomes that were recorded before discontinuation were included as covariates. For a pre-selection of predictors, a statistical learning approach (LASSO regression) was applied.

Hypothesis testing was conducted by a comparison of changes in the IG and the CG regarding the primary and secondary outcomes. The primary confirmatory analysis of the study was the comparison of mean changes in the primary outcome of function, measured by the Hannover Functional Ability Questionnaire (HFAQ, 1–100 [46]) at 12 months (t4).

Therefore, a saturated 4 × 2-factorial linear mixed effects model was fitted for primary and secondary outcomes, including all four measurement time points. Both groups were included as fixed effects and intercept and slope were additionally included as random effects whilst controlling for statistically significant differences at baseline [67]. Because of the disparity between the duration of the inpatient rehabilitation phase (27 days on average) and the follow-up phase (12 months), separate slopes for these phases were estimated by a spline model. Primarily, overall group differences were identified by a model comparing a likelihood ratio test of a hypothesised model incorporating the interaction effects of group by time and a nested null model leaving out these interactions.

The data analysis followed the principles of the ‘intention-to-treat’ approach [68].

For the primary outcome of function and the secondary outcome of pain, we examined the proportion of participants reaching a minimal clinically meaningful level of improvement based on the full available data at baseline and after 12 months. Based on Ostelo et al. [69], a minimal clinically meaningful improvement was defined as a 30% reduction from the baseline score.

Regarding within-group effects, standardised effect sizes (SES) were reported for each study group. The statistician was blinded to the group allocation.

## Results

### Flow chart (Fig. 1)

**Fig. 1:**
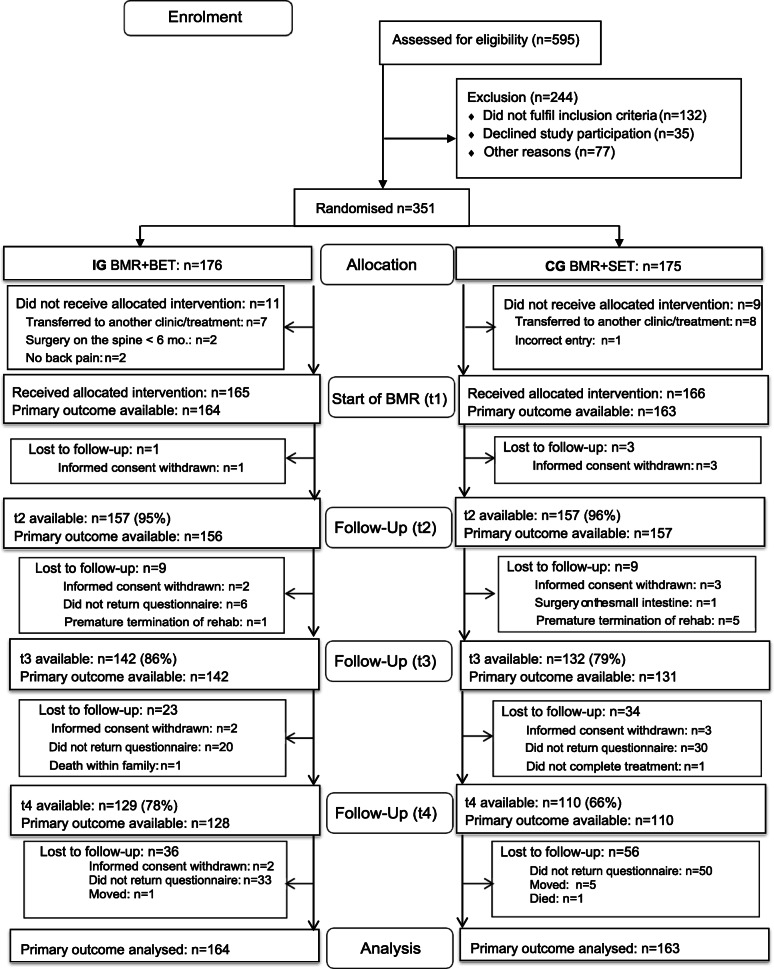
Flow chart. t1 = start of BMR; t2 = end of BMR; t3 = 6 months; t4 = 12 months

### Description of the study sample

The initial sample included 327 participants with CLBP (CG: *n* = 163; IG: *n* = 164). Table 2 shows the baseline characteristics of all participants by study group. Participants were mainly female (79%), and the mean age was 51 years (SD: 7,4). No significant differences between participants in both study groups were found regarding most baseline characteristics.

Of 595 participants who had been assessed for eligibility, 351 signed the informed consent and were randomly assigned to the CG or IG.

A total of 244 participants had been excluded due to various reasons (see Fig. 1). The dropout rate throughout the study was 27,8% on average and was higher in the CG (t2: 5,4%; t3: 20,5%; t4: 33,8%) compared to the IG (t2: 4,8%; t3: 13,9%; t4: 21,8%) at the end of the BMR and then after six and 12 months. The allocation to the CG was a significant predictor for the risk to drop out of the study at 12 months, with an odds ratio of 1.14 (95%KI: [1.04;1.26]). Unmarried participants with a partnership compared to unmarried participants without a partnership had a significantly lower risk of dropping out of the study. Other predictors, which reduced the risk of dropout, were cognitive attitudes towards physical activity and a longer duration of the BMR. In contrast, higher values for anxiety (GAD-7) slightly increased the chance of dropout. The explained variance of the overall model was low (Nagelkerkes R2 = 16.1%, Brier Score: 17.1%).

### Primary outcome

#### Functional ability (HFAQ)

BMR + BET was not superior with regard to improving function compared with BMR + SET at the end of the BMR (t1-t2) (mean difference, 0.08; 95% CI − 2.82 to 2.99; *p* = 0.955), in the phase after the BMR (t2-t4) (mean difference, − 1.80; 95% CI -1.80; − 5.57 to 1.97; *p* = 0.349) and after 12 months (t1-t4) (mean difference, − 1.33; 95% CI − 5.57 to 2.92; *p* = 0.540). Both programmes, BMR with BET and BMR with SET, significantly improved function over the course of 12 months with small effect sizes. See Table 3 for the mean changes (SDs) in both study groups in each phase.

**Table 3:** Functional ability (HFAQ)

Functional Ability								
Phase	BMR + SET Mean change (SD)	SES	***p***-value	BMR + BET Mean change (SD)	SES	***p***-value	adjusted mean difference (95% CI)	***p***-value
t1-t2	5.85 (13.19)	0.44	**0.000**	5.94 (13.09)	0.45	**0.000**	0.08 (−2.82 to 2.99)	0.955
t2-t4	0.37 (16.09)	0.02	0.794	−1.44 (15.72)	−0.09	0.278	−1.80 (− 5.57 to 1.97)	0.349
t1-t4	6.02 (18.06)	0.33	**0.000**	4.69 (17.74)	0.26	**0.002**	−1.33 (− 5.57 to 2.92)	0.540

*BMR* behavioural medical rehabilitation, *SET* standard exercise therapy, *BET* behavioural exercise therapy, *SES* standardised effect size, *SD* standard deviation, *CI* confidence interval, *t1* start of BMR, *t2* end of BMR, *t3* 6 months, *t4* 12 months, *p* significance Level, *bold p* < 0.05; italic = *p* < 0.10.

A minimal clinically meaningful improvement of 30% [69] in function based on the full available data at baseline and after 12 months showed 13 participants in the BMR + SET group and 17 participants in the BMR + BET group. A comparison between BMR + SET and BMR + BET showed further 0 versus 3 participants experienced a decline in function, and 109 versus 97 reported no change, respectively.

### Secondary outcomes

There were no significant between-group differences in pain intensity (NRS) at the end of the BMR (t1-t2), in the phase after the BMR (t2-t4) and after 12 months (t1-t4).

In behavioural pain coping strategies (FESV), there was a significant between-group difference in the use of counter activities in favour of the BMR + BET group at the end of the BMR (t1-t2). Regarding other behavioural pain coping strategies, such as mental distraction and relaxation, no significant between-group differences were identified. There were also no significant between-group differences in cognitive pain management strategies (FESV) with action-oriented coping, cognitive restructuring, and subjective coping competence.

The same was the case for the following secondary outcomes: health-related quality of life (physical and mental composite scale) (SF 12), depression (PHQ-D), anxiety (GAD 7), stress (PSS), kinesiophobia (TSK), avoidance-endurance (AEQ), physical activity (FFkA), emotional, motivational, and volitional determinants of physical activity. A significant between-group difference was found for action planning at the end of the BMR (t1-t2).

Both programmes, BMR with BET and BMR with SET, significantly improved most of the secondary outcomes with small to medium within-group changes.

All results and the unadjusted means (SDs) for the primary and secondary outcomes are available in Additional file 2.

## Discussion

BMR with BET did not lead to superior improvements in function for participants with CLBP in comparison to BMR with SET. It had been hypothesised that a behavioural approach to exercise therapy [12], which addresses both relevant determinants of pain chronification [34–36] and determinants of physical activity behaviour change [29, 30, 32], improves the long-term effectiveness of BMR compared to BMR with SET.

The analysis of secondary outcomes, such as pain, anxiety, depression, quality of life, stress, pain management, coping with pain, physical activity and the motivational/volitional determinants of physical activity also did not yield significant between-group differences. Both programmes, BMR with BET and BMR with SET, significantly improved function over the course of 12 months with small effect sizes. This is also the case for secondary outcomes with some exceptions, which either yielded no improvements or where the within-group changes amounted to small to medium effect sizes.

A systematic variation of single treatment components within a complex rehabilitation programme is one way to better understand the underlying mechanisms of action of these multi-component interventions and to improve our knowledge about which specific treatment components might improve treatment efficacy [18]. The focus in our study was on the component of exercise therapy within in the BMR: the effects of a behavioural approach (BET), compared to a biomedical approach (SET), on the effectiveness of BMR.

Concerning the present study, it can be critically discussed whether the conceptual differences between BET and SET within the BMR were sufficient to reach a different effect on the primary outcome after 12 months. Both study groups received a complex BMR with the aim to improve self-management of CLBP and around 65 h of therapy including 26 h of exercise therapy in approximately 4 weeks. In the following sections we will discuss the conceptual overlap or differences between both exercise programmes along the three aims of BET. This approach systematically combined exercise with patient education and behavioural techniques to a) improve health-related fitness, b) promote a physically active lifestyle, and c) develop self-management of CLBP.

### Improvement of health-related fitness

Conceptually, both BET and SET contained a similar dose of exercise (e.g. general resistance training, water-based therapy, walking/Nordic walking and stationary cycling) of approximately 26 h over a four-week period. Although clear dose-response relationships have not yet been established for exercise in CLBP, this dose of exercise in SET and BET seemed sufficient to expect at least first acute physiological adaptions. This in turn may have contributed to the observed increase in function in both study groups after 4 weeks corresponding to a medium effect size.

Exercise influences pain and function in CLBP by potentially 33 unique mechanisms, but a clear understanding of why it works has not yet been established [15]. These mechanisms were grouped into five themes by Wun et al. [15] of which four, namely neuromuscular, neurophysiological, cardiometabolic, and tissue healing, were likely influenced by BET as well as SET. However, there is only preliminary evidence how exercise-related biopsychosocial mechanisms might mediate changes in function and pain in individuals with CLBP [70].

BET and SET where both embedded into a biopsychosocial BMR and it was not possible in this study to identify a unique mechanism which is only present in BET or SET. Based on the biomedical approach in SET and the biopsychosocial approach in BET, it is likely that psychosocial mechanisms were specifically addressed in BET while other mechanisms (e.g. neuromuscular, neurophysiological, cardiometabolic, tissue healing) were addressed in SET as well as BET. Despite the possible existence of a unique mechanism, explaining a major proportion of the variance in function and pain, no particular mode of exercise has proven to be superior to other modes in CLBP and other chronic pain conditions [4, 18, 71]. Most of these studies compare two types of exercise interventions designed to improve physical fitness rather than self-management and physical activity promotion in CLBP.

Future studies could investigate how exercise mediates changes in function and pain in individuals with disabling CLBP who have not responded well to other treatments, and whether addressing these mechanisms improves the effectiveness of exercise therapy. This requires study designs that allow a unique mechanism to be identified and where exercise is assessed as a single component rather than embedded in a complex programme like BMR.

### Promotion of a physically active lifestyle

A physically active lifestyle is associated with numerous health benefits and is also recommended for people living with a chronic disease by the WHO [72]. There is evidence, that a physically active lifestyle protects against the incidence of low back pain [73], and is associated with a lower prevalence of low back pain [74]. Low levels of physical activity were also associated with the development of disabling CLBP, although uncertainties remain regarding independent associations [75].

Reviews have shown that behaviour change techniques might improve exercise adherence in various populations with chronic conditions, including CLBP [31, 32]. More recent reviews show that supervised exercise programmes with motivational strategies [76, 77] as well as pain science education, graded exposure and multimodal interventions [78] improve exercise adherence in individuals with CLBP, while action plans or self-management were not supported [76, 78].

BET included several behaviour change techniques to address motivational and volitional determinants of physical activity behaviour change (see https://bit.ly/3u69mwz). In contrast, the biomedically oriented SET did not contain any behaviour change techniques to systematically promote exercise adherence. In line with this, significant between-group differences were to be expected for the motivational/volitional determinants of physical activity as well as the corresponding physical activity behaviour. Only participants from the IG reported a significant increase in self-reported sport activity of 53 min per week after 12 months, compared to the start of the BMR, whereas participants in BMR + SET performed on average 22 min less sport activity. In our view, this is a relevant finding, as even small increases of physical activity are associated with health benefits and are recommended by the recently published WHO Guidelines on physical activity and sedentary behaviour [72]. Overall, there were no significant between-group differences regarding physical activity.

Motivational/volitional determinants and physical activity were already quite high in both study groups at the start of the BMR and additional improvements could not be expected. Determinants of motivation and volition are not taken into account as selection criteria for assigning participants to BET within the BMR. Considering a more person-centred and targeted approach to exercise therapy [29], the inclusion of motivational or volitional determinants as selection criteria for assigning participants to a BET within a BMR could improve effectiveness in promoting a physically active lifestyle.

### Development of self-management strategies to deal with CLBP

According to cognitive-behavioural models [34, 36, 43], very high or very low levels of physical activity represent maladaptive pain coping behaviours as a consequence of maladaptive emotions and beliefs. Exercise therapy with a cognitive-behavioural approach [11, 79], such as graded activity, addresses psychosocial mechanisms more specifically by altering maladaptive pain-related beliefs, emotions and behaviour. Currently, there is limited evidence for a long-term improvement in function when graded activity is compared to a CG, and no difference has been found when it is compared to other exercise interventions [80].

The physical therapists in BET used patient education and focused on beliefs, emotions, and behaviour in relation to physical activity. For example, they discussed positive physiological health-related effects of physical activity and its psychological benefits (e.g. stress and mood management), the relevance of maladaptive pain-related beliefs and behaviour (e.g. avoidance or endurance behaviour), as well as adaptive responses (e.g. physical activity for relaxation or mood management). They further introduced different types of exercise, as mentioned above, and encouraged participants to practice physical activity that is in line with their experiences and individual preferences. Physical therapists introduced the “Rate of Perceived Exertion”-Scale (Borg scale, ranging from 6 to 20) and encouraged participants to exercise at a level that is ‘somewhat hard’ [81]. Additionally, participants were trained in manual pulse measurement to control intensities of aerobic exercises. Further details regarding the core principles, methods and contents of BET are available in the manual (see https://bit.ly/3u69mwz), in the study protocol [12], as well as in the Additional file 1.

One could argue that addressing maladaptive beliefs, emotions, and behaviour in persons with CLBP is not within the scope of the physical therapy profession or that therapists are not trained well enough accordingly. There is a broad range of challenges when psychosocial principles ought to be integrated into physical therapist practice, but many might be overcome with appropriate training [82, 83]. Nicholas et al. [26] have outlined the skills for physical therapists to perform psychologically informed treatments, which are also supported by the recent curriculum for physical therapy of the IASP (https://bit.ly/2PcNXDk). In our study all physical therapists who delivered BET had been trained intensively and had been provided with a very detailed manual with additional material to support the implementation.

SET did not contain specific patient education or cognitive-behavioural techniques to systematically alter maladaptive beliefs, emotions, or behaviours in CLBP. The physical therapists in SET delivered education about anatomy and physiology of the spine, pathophysiological causes of CLBP and posture/movement during activities of daily living. However, it is possible that SET within BMR influences psychological risk factors such as pain-related beliefs, emotions and behaviour to some extent without using specific content or techniques to address these factors (e.g. [84, 85]. If this is the case, the similar trajectories in the primary outcome as well as the secondary outcomes in both study groups might have been the consequence.

Within BMR, both study groups received a psychological treatment with six to eight sessions of around 60 min, which had also a focus on self-management of CLBP [7, 12]. The psychological therapists in the BMR brought up the role of beliefs, emotions and behaviour in coping with pain but did not focus specifically on physical activity.

In BET there was a clear focus on self-management of CLBP in relation to physical activity [12]. Considering a recently published review [86], the BET approach might rest too narrowly on certain psychological dimensions, whereas social factors (e.g. work, family, relationships, socioeconomic status, environment) are not addressed in either SET or BET. However, some of these social factors are addressed within the other components of the BMR (e.g. social counselling, psychological treatment). This biopsychosocial approach of the BMR was applied in both study groups, which likely contributed to the similar effects in both of them.

In summary, there is a conceptual difference between BET and SET within the BMR as the described aims, methods and contents in BET to promote a physically active lifestyle as well as to promote self-management of CLPB were only provided in the BMR + BET study group and not in the BMR + SET study group.

In the following sections we discuss other possible reasons for the observed results, compare these with other studies in the field and discuss strengths and limitations of our study.

### Quality of the implementation of BET

A potentially low quality of the implementation of BET could be another explanation for the observed results. It cannot be ruled out entirely that important modifications from the BET manual may have taken place as it was not possible to supervise all BET sessions during the study period and visits had to be announced. We did not identify modifications from the written BET manual during our two announced visits. We could not detect any changes which might be a sign for administrative challenges for the delivery of BET during the study period. Therefore, there is no evidence of a low-quality implementation of BET.

### Comparison with other studies

We identified seven RCTs [6, 87–92] and four quasi-experimental studies [61, 93–95] that compared two or more rehabilitation programmes in participants with CLBP in a setting similar to that of our study. Three of the RCTs [6, 90, 91] used function as a primary outcome, and one [92] as a secondary outcome. The measurement differed as the Oswestry Disability Index was used three times as the primary outcome measure [89–91], the Pain Disability Index once [6] and the Hannover Functional Ability Questionnaire once [92]. Most of these RCTs had a 12-month follow-up [6, 90, 91], one had 6 months [92] and one had 12 weeks [89]. Only two of these studies [89, 91] assessed different approaches of exercise therapy but showed no between-group differences at 12 weeks [89] or 12 months [91] in function, which is in line with our study results. Iversen and colleagues [89] compared two rehabilitation programmes that differed only in the type of standardised exercise therapy used (general exercise versus resistance band training), while Verra et al. [91] compared two rehabilitation programmes of which one used tailored interventions by matching subgroups with specific pain profiles. These tailored interventions included physiotherapy/exercise therapy based on a behavioural approach. Neither study included behaviour change techniques to foster a physically active lifestyle as used in BET [12].

One RCT [6] compared a traditional multidisciplinary rehabilitation with a biopsychosocial one. It [6] showed no between-group differences in function at a 12-month follow-up. The exercise therapy component in this RCT was based on a biomedical approach.

In summary, the comparability of these RCTs [6, 89–92] to our study is limited due to different measurements, study designs, follow-ups, and heterogeneous interventions that differ in content and dose. This challenge has also been described recently by Schmidt et al. [90]. However, in line with our results, these RCTs did not show a between-group difference in function at follow-up.

The remaining above-mentioned RCTs [87, 88] compared two nearly identical traditional multidisciplinary rehabilitation programmes but with one including an optimised treatment component (e.g. back school, psychological treatment) and reported results favouring the rehabilitation programme with the optimised component with corresponding small effect sizes. They used other primary outcome measures and did not report function as the primary outcome even though the measurement of function has been established as a core outcome measure [59, 60]. This limits the comparability of these RCTs with our study.

In our previous quasi-experimental study [61], we showed a small between-group difference in function after 12 months favouring a multidisciplinary biopsychosocial rehabilitation that included the same BET approach which was used in the current study, compared to a traditional multidisciplinary rehabilitation with SET. Due to the comparison of two different complex rehabilitation programmes, it was not possible to analyse how BET might have contributed to the observed between-group difference in this study. The other quasi-experimental studies which were conducted in the German rehabilitation setting in study populations with CLBP and other musculoskeletal disorders [93–95] did not report function as outcome measures which limits the comparability with our current study. All quasi-experimental studies included cognitive-behavioural strategies in line with a biopsychosocial framework in the intervention groups and reported favouring results in comparison to a traditional multidisciplinary rehabilitation with a biomedical approach. However, it must be taken into account that quasi-experimental designs increase the risk of bias compared to an RCT design and have other methodological limitations.

### Study population in BMR

Another explanation for the observed results relates to the study population in the BMR. Multidisciplinary biopsychosocial rehabilitation is recommended for individuals with CLBP and associated disability [4, 5]. It has been stated in a recent review [5] that higher symptom severity might modify the effect of multidisciplinary biopsychosocial rehabilitation even though this could not be supported in subgroup analyses as only a few studies (3 of 41) included a study population with a higher baseline severity defined by 60% of the maximum score on a pain and a function measure. In our study, the percentage of participants with a strong functional decline (> 60%) at baseline was 41%, and the percentage of those with high pain levels (> 7 points on the numeric scale) was 34%. Furthermore, a 30% improvement in function and pain is considered a clinically meaningful change in participants with CLBP [69, 96]. Such an improvement was detected in 11% of the CG (*n* = 13) and 15% of the IG (*n* = 17) for function as well as 19% of the CG (*n* = 20) and 18% of the IG (*n* = 22) for pain. Overall, both study groups showed only a moderate functional decline and pain intensity at baseline, which might have reduced the probability of improvements on an absolute scale. In other secondary outcomes, the study participants were also only slightly or moderately impaired, and the percentage of participants with strong impairments was below 40% in both study groups. Future research should further explore what works for whom and which subgroup might benefit the most from a complex BMR. This would improve the effectiveness of this treatment in the long-term.

### BMR, BET and mechanism-based treatment options

BMR is a group-based intensive treatment option for participants with disabling CLBP, who have not responded to other treatment options. Our results show that BET+BMR as well as SET+BMR improve function, pain, and other secondary outcomes in participants with disabling CLBP. However, not all participants with disabling CLBP benefit equally from a BMR. The challenge within a group-based BMR is to consider the heterogeneity in disabling CLBP with individual mechanisms that might contribute to CLBP and to address this with a correspondingly arranged treatment plan. The concept of BMR [7] takes individual mechanisms into account, for example with multidisciplinary admission, standardised psychosocial assessment, reconciled multidisciplinary case management, case reviews on a regular basis, and the possibility of an individually tailored therapy schedule. The concept also puts a strong emphasis on individual preferences and experiences of the participants with CLBP.

Another widespread approach in pain management assumes that individuals with CLBP benefit more from an individualised treatment option that targets a specific underlying pain mechanism [97, 98]. The idea of a mechanism-based classification of pain dates back more than 20 years [99], but for the majority of those affected with non-specific disabling CLBP it doesn’t seem to be possible to identify a specific underlying mechanism [9, 100]. On the one hand, group-based interventions in the treatment of disabling CLBP have been criticized as less effective due to their one-size-fits-all approach, on the other hand, a rigid definition of mechanism-based subgroups based on a mostly unidimensional pathophysiological rationale was also criticized [98, 101, 102]. Such a mechanism-based classification is particularly challenging in disabling CLBP as multiple factors of different dimensions often interact with one another [100].

Novel approaches such as multidimensional clinical reasoning take into account the individual context as well as the complex interplay between multiple factors that could influence function and pain in participants with CLBP [101]. O’Keeffe et al. [103] compared such an individualised cognitive functional therapy (CFT) with a group-based exercise and education intervention and reported reduced disability, but not pain, at 6 and 12 months in favour of CFT. However, Mescouto et al. [86] have described that although CFT is based on a biopsychosocial approach, the focus is on changing individual cognitions and behaviour, but less on other social factors in the wider social context that might also contribute to disabling CLBP.

To the best of our knowledge, there are no studies to date on how an individual mechanism-based approach of exercise therapy influences the effectiveness of a multidisciplinary biopsychosocial rehabilitation, such as the BMR, in the treatment of disabling CLBP. Based on our results in this study, we speculate that only a small between-group difference could be expected. In addition, from a cost-benefit perspective, it is currently not known how an individual mechanism-based approach in exercise therapy compares to an intensive group-based multidisciplinary biopsychosocial rehabilitation. The best available moderate to low quality evidence to date shows that a multidisciplinary biopsychosocial rehabilitation is more effective than physical treatment or usual care in reducing disability and pain in participants with disabling CLBP [5]. Future studies could investigate whether optimized individual mechanism-based approach of exercise therapy, such as the CFT, in the treatment of CLBP are equally effective as a multidisciplinary biopsychosocial rehabilitation. The results would presumably have important consequences for future decision-making processes in the management of CLBP. To further improve our understanding of the underlying mechanisms of action of complex rehabilitation programmes, pre-planned mediation analyses within adequately powered RCTs that consider known mediators based on the biopsychosocial framework would also be helpful [104].

Our trial had several **strengths**. In both rehabilitation centres, an evidence-based BMR was conducted. Thereby, the study had a strong CG as we compared two intensive and complex multidisciplinary biopsychosocial rehabilitation programmes that differed only in one treatment component instead of a comparison with wait-list CGs, less complex interventions or usual care. The study was registered prospectively and incorporated an external, blinded ‘urn’-randomisation [64, 65], which is a generalization of the class of biased-coin designs. It is a method designed for stratification while preserving randomness and basically avoids some of the disadvantages of stratified block randomisation. However, this randomisation algorithm cannot be described in terms of simple randomisation algorithms. The probability that a subject will be assigned to a given treatment group is not constant. It is systematically biased in favour of balance, but not as strong as e.g. block randomisation towards the end of every block, where allocation gets deterministic in trade off for balance. Further strengths of this study are the manualised BET, an intention-to-treat analysis and concealed allocation. The statistical analysis was blinded and was performed by a statistician who did not directly take part in conducting the study. The study was funded independently and publicly.

Several **limitations** have to be taken into account. Dropout after six and 12 months was higher in the CG (21, 34%) compared to the IG (14, 22%). Even though reduced function at baseline does not seem to be a relevant predictor for dropout at the last point of measurement, it cannot be ruled out entirely that participants whose function deteriorated later did dropout more often in the CG. Data not missing at random (NMAR) might have taken place.

The statistical power was calculated to detect a medium effect in the between-group difference of the primary outcome. As we compared two highly intensive multidisciplinary biopsychosocial rehabilitation programmes which differed only in the approach of exercise therapy, even a small between-group difference would have been of interest. We therefore decided to increase the number of participants in the study sample in the available study period. We made this decision early in the course of the study, before we started any preliminary data analysis. However, the final study sample was not sufficient to detect a very small between-group difference. Given the high burden of CLPB on individuals and the society, even a small effect would be important.

The blinding of the study’s therapists was not possible. Even though visitations did not indicate a contamination, this might have happened at some point during the intervention period lasting for 12 months.

The focus of this study was on the influences of a behavioural approach to exercise therapy on the effectiveness of a BMR. In line with the aim of the study we assessed psychosocial determinants as well as determinants of health behaviour change in addition to recommended core outcome domains [59, 60]. A valid assessment of health-related fitness at all four measurement time points requires equipment and additional personnel and financial resources which were not available.

BMR in Germany is a complex and intensive process based on a comprehensive and consistent biopsychosocial framework that includes key elements which characterise an effective rehabilitation as described by Wade et al [41]. Our results indicate that SET as well as BET, when embedded in such a BMR approach, are both equally effective. It seems reasonable to assume that the observed effects in both study groups, which showed a similar course in the primary as well as most of the secondary outcomes with small-to-medium effect sizes in both study groups, are influenced by similar biopsychosocial mechanisms. However, this needs to be further explored in future studies as a similar effect does not necessarily have to be caused by the same underlying mechanisms.

Taking a wider perspective into account [90], CLBP and associated disability is a major public health problem, and no single intervention or solution will guide its prevention and management [4, 9, 18]. Sustaining long-term improvements in individuals with persistent low back pain and reducing the societal burden of CLBP requires embedding the intensive BMR into a whole-of-society approach, where ‘local participation and ownership, integration with existing priorities and policies, and coordination with national and regional systems and processes are crucial’ [105], p 2386).

## Conclusion

SET+BMR and BET+BMR do not differ in terms of their effectiveness in improving function or other secondary outcomes. Therefore, a SET with a biomedical approach, when embedded into a complex BMR, is equally effective compared to BMR with a biopsychosocial orientated BET. However, within BMR, in contrast to SET, BET offers a standardised, manualised, evidence-based and theory-based approach, which is recommended for the BMR by the German Pension Insurance Association. Implementing BET in multidisciplinary biopsychosocial rehabilitation such as BMR for the treatment of CLBP is one option in routine care. There are only few studies available which have studied the effects of a biopsychosocial approach to exercise therapy on the effectiveness of a complex inpatient rehabilitation programme. Future studies in this area of research would further contribute to an improved understanding of the mechanisms of action of multidisciplinary biopsychosocial rehabilitation. These studies should consider pre-planned and well powered mediation analyses as well as mechanism-based and person-centred approaches to exercise therapy which take into account the biopsychosocial framework in the development of CLBP.

## Supplementary Information

**Additional file 1.** Description of the 15 units of BET (closed group) with an overview of contents: education about CLBP, education about physical activity, coping with pain, and exercises.

**Additional file 2.** Tables with results for all secondary outcomes. Unadjusted means for the primary outcome and each secondary outcome at t1 = start of BMR, t2 = end of BMR, t3 = 6 months; t4 = 12 months for each study group.

## Data Availability

The datasets used and/or analysed in the current study are available from the corresponding author on reasonable request.

## Abbreviations

BMR: Behavioural medical rehabilitation
CLBP: Chronic non-specific low back pain
BET: Behavioural exercise therapy
SET: Standard exercise therapy
RCT: Randomised controlled trial
IG: Intervention group
CG: Control group
HFAQ: Hannover Functional Ability Questionnaire
NRS: Numeric Rating Scale
PHQ-D: Patient Health Questionnaire
GAD-7: General Anxiety Disorder 7-item Scale
SF-12: Short-Form-12
PSS: Perceived Stress Scale
FFkA: Freiburg Questionnaire on Physical Activity
FESV: Pain Management Questionnaire
TSK: Tampa Scale for Kinesiophobia
AEQ: Avoidance-Endurance Questionnaire
SES: Standardised effect size
SD: Standard deviation
ICD 10: International Classification of Diseases 10
CI: Confidence interval
NMAR: Not missing at random
