# Supplementary material for: Effects of behavioural exercise therapy on the effectiveness of multidisciplinary rehabilitation for chronic non-specific low back pain: a randomised controlled trial
Source: BMC Musculoskelet Disord. 2021 May 29;22:500. doi: 10.1186/s12891-021-04353-y (PMC8164753; doi:10.1186/s12891-021-04353-y)
Supplement: Supplementary file 1 — Additional file 1. Description of the 15 units of BET (closed group) with an overview of contents: education about CLBP, education about physical activity, coping with pain, and exercises. [file 12891_2021_4353_MOESM1_ESM.pdf]

Behavioural exercise therapy (BET) 15 sessions, closed group

Session	1	2	3	4	5	6	7	8	9	10	11	12	13	14	15
BET closed group 60min	Introduction	Introduction, review of last session	Introduction, review of last session	Introduction, review of last session	Introduction, review of last session	Introduction, review of last session	Introduction, review of last session	Introduction, review of last session	Introduction, review of last session	Introduction, review of last session	Introduction, review of last session	Introduction, review of last session	Introduction, review of last session	Introduction, review of last session	introduction, review of last session
	Active play: get to know each other	Active play: get to know each other	Active play: get to know each other	Active play: positive exercise experiences	Active play: positive exercise experiences	Active play: positive exercise experiences	Active play: positive exercise experiences	Active play: positive exercise experiences	Active play: positive exercise experiences	Active play: positive exercise experiences	Active play: positive exercise experiences	Active play: positive exercise experiences	Active play: positive exercise experiences	Active play: positive exercise experiences	Active play: positive exercise experiences
	What is low back pain (lbp)? Prevalence and course (acute, subacute, chronic lbp)	Why does lbp persist? Lack of causes, possibilities and limitations of diagnostic procedures	The spine as a fascinating and stable system	Effects of physical activity on the whole organism. "Physical activity is good for your body"	Effects of physical activity on mood and well-being. Stress and mood management	"Coper, fear-avoider, endurer": strengthening the pain defense system	Enjoyment through physical activity and exercise	Education: muscular stabilization of the spine: How does it work?	Education (repetition): muscular stabilization and use of it during activities of daily living	Physical activity in everyday life	Repetition: lumbar stabilization exercises during everyday movements	Behaviour during recurring pain episodes ("flare ups"); avoidance/endurance (repetition)	Action planning for future maintenance of physical activity: "goals and plans (when, where, how)"	Coping planning for future maintenance of physical activity: "barriers and barrier management"	Information about aftercare and support for contacting respective providers
	Active break: gymnastic elements	Active break: gymnastic elements	Body awareness exercise „the human pendulum"	Active play ("ball transportation")	Active break: gymnastic elements		Active play: positive exercise experiences	Introduction of lumbar stabilization exercises: activation of deep and global trunk muscles	Lumbar stabilization exercises: activation of deep and global trunk muscles during activities of daily living	Self-directed functional gymnastics *(standard program)*	Education: lumbar stabilization during work related physical activities				Alternative exercises "Qi Gong"
	Causes and risk factors regarding lbp	Why does lbp persist? Consequences of pain, pain memory and positive effects of physical activity	Self-directed functional gymnastics: introduction of additional exercises and training control strategies	Dealing with lbp: passive and active self-management strategies	Effects of physical activity on the interplay of pain and mood	Self-directed functional gymnastics *(standard program)*	Thoughts, moods and posture (photo story)	Self-directed functional gymnastics *(standard program)*	Variation of the body awareness exercise „human pendulum"		One sided loads/strain and compensation strategies	Alternative exercises "Qi Gong"			
	One sided load/strain versus movement	Self-directed functional gymnastics: introduction of additional exercises and training control strategies	Metaphorical story "mushroom picker"	Self-directed functional gymnastics *(standard program)*	Self-directed functional gymnastics *(standard program)*		Self-directed functional gymnastics *(standard program)*		Self-directed functional gymnastics *(standard program)*	Alternative exercises "Qi Gong"	Alternative exercises "Qi Gong"		Alternative exercises "Qi Gong"	Alternative exercises "Qi Gong"	Final group discussion and "take home messages"
	Self-directed functional gymnastics (strengthening and stretching exercises). Introduction of 2 exercises and training control strategies		Relaxation			Relaxation		Relaxation							
	Conclusion	Conclusion and metaphorical story "Hiking"	Conclusion	Conclusion	Conclusion	Conclusion	Conclusion	Conclusion	Conclusion	Conclusion	Conclusion	Conclusion and metaphorical story "Has everything been done?"	Conclusion	Conclusion	Conclusion

Education about low back pain (lbp)
Exercise/ training/ relaxation
Coping with low back pain (lbp)
Education about effects of physical activity and exercises
