# Supplementary material for: Effects of behavioural exercise therapy on the effectiveness of multidisciplinary rehabilitation for chronic non-specific low back pain: a randomised controlled trial
Source: BMC Musculoskelet Disord. 2021 May 29;22:500. doi: 10.1186/s12891-021-04353-y (PMC8164753; doi:10.1186/s12891-021-04353-y)
Supplement: Supplementary file 2 — Additional file 2. Tables with results for all secondary outcomes. Unadjusted means for the primary outcome and each secondary outcome at t1 = start of BMR, t2 = end of BMR, t3 = 6 months; t4 = 12 months for each study group. [file 12891_2021_4353_MOESM2_ESM.docx]

### Pain intensity (NRS)

| ***Table 1*: Pain Intensity (NRS)** | | | | | | | | |
| --- | --- | --- | --- | --- | --- | --- | --- | --- |
| **Phase** | **BMR+SET Mean change (SD)** | **SES** | ***p*-value** | **BMR+BET Mean change (SD)** | **SES** | ***p*-value** | **adjusted mean**  **difference (95% CI)** | ***p*-value** |
| t1-t2 | -5.38 (14.8) | -0.36 | **0.000** | -5.52 (14.63) | -0.38 | **0.000** | -0.14 (-3.4 to 3.12) | 0.933 |
| t2-t4 | -10.45 (18.94) | -0.55 | **0.000** | -8.67 (18.57) | -0.47 | **0.000** | 1.79 (-2.67 to 6.24) | 0.433 |
| t1-t4 | -13.13 (22.31) | -0.59 | **0.000** | -12.09 (22.03) | -0.55 | **0.000** | 1.04 (-4.22 to 6.31) | 0.698 |

BMR=behavioural medical rehabilitation; SET=standard exercise therapy; BET=behavioural exercise therapy; SES=standardised effect size; SD=standard deviation; CI=confidence interval; t1=start of BMR; t2=end of BMR; t3=6 months; t4=12 months; p=significance Level; bold=p<0.05; italic=p<0.10

###

### Pain management (FESV)

| ***Table 2*: Cognitive and behavioural pain management strategies (FESV)** | | | | | | | | |
| --- | --- | --- | --- | --- | --- | --- | --- | --- |
| **Phase** | **BMR+SET Mean change (SD)** | **SES** | ***p*-value** | **BMR+BET Mean change (SD)** | **SES** | ***p*-value** | **adjusted mean**  **difference (95% CI)** | ***p*-value** |
| Action-oriented coping | | | | | | | | |
| t1-t2 | 2.60 (4.12) | 0.63 | **0.000** | 3.19 (4.16) | 0.77 | **0.000** | 0.59 (-0.35 to 1.53) | 0.222 |
| t2-t4 | -1.06 (4.86) | -0.22 | **0.013** | -1.33 (4.77) | -0.28 | **0.001** | -0.27 (-1.41 to 0.88) | 0.647 |
| t1-t4 | 1.74 (5.16) | 0.34 | **0.000** | 2.08 (5.15) | 0.40 | **0.000** | 0.34 (-0.88 to 1.57) | 0.584 |
| Cognitive restructuring | | | | | | | | |
| t1-t2 | 3.14 (4.15) | 0.76 | **0.000** | 3.57 (4.19) | 0.85 | **0.000** | 0.43 (-0.52 to 1.38) | 0.377 |
| t2-t4 | -1.28 (4.95) | -0.26 | **0.004** | -2.22 (4.85) | -0.46 | **0.000** | -0.94 (-2.11 to 0.22) | 0.114 |
| t1-t4 | 2.18 (5.30) | 0.41 | **0.000** | 1.63 (5.27) | 0.31 | **0.000** | -0.55 (-1.81 to 0.71) | 0.392 |
| Subjective coping competence | | | | | | | | |
| t1-t2 | 1.74 (3.82) | 0.46 | **0.000** | 2.29 (3.85) | 0.60 | **0.000** | 0.55 (-0.32 to 1.43) | 0.217 |
| t2-t4 | -1.08 (4.38) | -0.25 | **0.005** | -1.24 (4.31) | -0.29 | **0.001** | -0.16 (-1.19 to 0.87) | 0.763 |
| t1-t4 | 0.91 (4.53) | 0.20 | **0.022** | 1.27 (4.51) | 0.28 | **0.001** | 0.35 (-0.72 to 1.43) | 0.517 |
| Mental distraction | | | | | | | | |
| t1-t2 | 2.27 (4.11) | 0.55 | **0.000** | 2.41 (4.14) | 0.58 | **0.000** | 0.14 (-0.8 to 1.09) | 0.764 |
| t2-t4 | -2.39 (4.79) | -0.50 | **0.000** | -2.19 (4.72) | -0.46 | **0.000** | -0.21 (-0.92 to 1.34) | 0.719 |
| t1-t4 | 0.12 (5.03) | 0.02 | 0.779 | 0.42 (5.01) | 0.08 | 0.317 | 0.30 (-0.89 to 1.49) | 0.621 |
| Counter activities | | | | | | | | |
| t1-t2 | 0.81 (3.77) | 0.22 | **0.010** | 1.71 (3.80) | 0.45 | **0.000** | 0.9 (0.04 to 1.76) | **0.042** |
| t2-t4 | -1.32 (4.36) | -0.30 | **0.001** | -1.26 (4.29) | -0.29 | **0.001** | 0.06 (-0.97 to 1.08) | 0.915 |
| t1-t4 | -0.47 (4.53) | -0.10 | 0.231 | 0.59 (4.51) | 0.13 | 0.119 | 1.07 (0.00 to 2.14) | *0.052* |
| Relaxation | | | | | | | | |
| t1-t2 | 3.88 (4.38) | 0.89 | **0.000** | 3.41 (4.41) | 0.77 | **0.000** | -0.46 (-1.47 to 0.54) | 0.365 |
| t2-t4 | -2.17 (4.96) | -0.44 | **0.000** | -1.89 (4.87) | -0.39 | **0.000** | 0.28 (-0.89 to 1.45) | 0.637 |
| t1-t4 | 2.03 (5.00) | 0.41 | **0.000** | 1.82 (4.95) | 0.37 | **0.000** | -0.20 (-1.39 to 0.98) | 0.734 |

BMR=behavioural medical rehabilitation; SET=standard exercise therapy; BET=behavioural exercise therapy; SES=standardised effect size; SD=standard deviation; CI=confidence interval; t1=start of BMR; t2=end of BMR; t3=6 months; t4=12 months; p=significance Level; bold=p<0.05; italic=p<0.10

### Fear avoidance/ Avoidance endurance responses (AEQ, TSK)

| ***Table* *3*: Avoidance/endurance (AEQ, TSK)** | | | | | | | | |
| --- | --- | --- | --- | --- | --- | --- | --- | --- |
| **Phase** | **BMR+SET Mean change (SD)** | **SES** | ***p*-value** | **BMR+BET Mean change (SD)** | **SES** | ***p*-value** | **adjusted mean**  **difference (95% CI)** | ***p*-value** |
| Depression | | | | | | | | |
| t1-t2 | -4.05 (3.81) | -1.06 | **0.000** | -4.04 (3.85) | -1.05 | **0.000** | 0.01 (-0.84 to 0.87) | 0.977 |
| t2-t4 | 2.57 (4. 62) | 0.56 | **0.000** | 2.10 (4.53) | 0.46 | **0.000** | -0.47 (-1.56 to 0.62) | 0.399 |
| t1-t4 | -1.72 (5.17) | -0.33 | **0.000** | -2.29 (5.12) | -0.45 | **0.000** | -0.57 (-1.80 to 0.65) | 0.360 |
| Anxiety | | | | | | | | |
| t1-t2 | -3.94 (3.72) | -1.06 | **0.000** | -4.39 (3.66) | -1.20 | **0.000** | -0.45 (-1.27 to 0.38) | 0.289 |
| t2-t4 | 2.35 (4.58) | 0.51 | **0.000** | 2.02 (4.49) | 0.45 | **0.000** | -0.33 (-1.41 to 0.75) | 0.553 |
| t1-t4 | -1.89 (5.22) | -0.36 | **0.000** | -2.65 (5.16) | -0.51 | **0.000** | -0.76 (-2.00 to 0.48) | 0.230 |
| Stress | | | | | | | | |
| t1-t2 | -1.99 (2.90) | -0.69 | **0.000** | -1.8 (2.89) | -0.63 | **0.000** | 0.19 (-0.45 to 0.83) | 0.565 |
| t2-t4 | 1.33 (3.48) | 0.38 | **0.000** | 0.58 (3.40) | 0.17 | **0.044** | -0.75 (-1.57 to 0.07) | *0.072* |
| t1-t4 | -0.78 (3.87) | -0.20 | **0.023** | -1.35 (3.79) | -0.36 | **0.000** | -0.57 (-1.48 to 0.34) | 0.217 |
| Catastrophising | | | | | | | | |
| t1-t2 | -0.17 (0.76) | -0.23 | **0.005** | -0.35 (0.75) | -0.46 | **0.000** | -0.17 (-0.34 to -0.01) | **0.044** |
| t2-t4 | 0.03 (0.93) | 0.03 | 0.750 | 0.23 (0.91) | 0.25 | **0.003** | 0.20 (-0.01 to 0.42) | *0.068* |
| t1-t4 | -0.15 (1.06) | -0.14 | 0.100 | -0.16 (1.04) | -0.15 | 0.075 | 0 (-0.25 to 0.24) | 0.971 |
| Help-/hopelessness | | | | | | | | |
| t1-t2 | -0.59 (0.95) | -0.62 | **0.000** | -0.75 (0.93) | -0.80 | **0.000** | -0.16 (-0.37 to 0.05) | 0.131 |
| t2-t4 | 0.07 (1.14) | 0.06 | 0.465 | 0.35 (1.12) | 0.32 | **0.000** | 0.28 (0.01 to 0.55) | **0.041** |
| t1-t4 | -0.56 (1.27) | -0.44 | **0.000** | -0.43 (1.25) | -0.35 | **0.000** | -0.13 (-0.17 to 0.43) | 0.398 |
| Thought suppression | | | | | | | | |
| t1-t2 | 0.02 (0.80) | 0.03 | 0.739 | 0.03 (0.80) | 0.04 | 0.634 | 0.01 (-0.17 to 0.19) | 0.921 |
| t2-t4 | -0.21 (0.91) | -0.23 | **0.008** | -0.31 (0.90) | -0.34 | **0.000** | -0.10 (-0.31 to 0.12) | 0.377 |
| t1-t4 | -0.19 (0.96) | -0.20 | **0.021** | -0.26 (0.94) | -0.27 | **0.001** | -0.06 (-0.29 to 0.16) | 0.582 |
| Anxiety/ depression | | | | | | | | |
| t1-t2 | -0.87 (1.03) | -0.85 | **0.000** | -0.77 (1.02) | -0.76 | **0.000** | 0.10 (-0.13 to 0.33) | 0.387 |
| t2-t4 | 0.57 (1.24) | 0.46 | **0.000** | 0.50 (1.21) | 0.41 | **0.000** | -0.06 (-0.13 to 0.13) | 0.667 |
| t1-t4 | -0.43 (1.39) | -0.31 | **0.000** | -0.34 (1.36) | -0.25 | **0.000** | 0.09 (-0.23 to 0.42) | 0.575 |
| Positive mood | | | | | | | | |
| t1-t2 | 0.67 (1.04) | 0.65 | **0.000** | 0.73 (1.02) | 0.72 | **0.000** | 0.06 (-0.16 to 0.29) | 0.579 |
| t2-t4 | -0.43 (1.23) | -0.35 | **0.000** | -0.55 (1.21) | -0.46 | **0.000** | -0.13 (-0.42 to 0.16) | 0.396 |
| t1-t4 | 0.34 (1.38) | 0.25 | **0.006** | 0.25 (1.35) | 0.19 | **0.027** | -0.08 (-0.41 to 0.24) | 0.613 |
| Avoidance of social activity | | | | | | | | |
| t1-t2 | -0.39 (0.93) | -0.42 | **0.000** | -0.35 (0.92) | -0.39 | **0.000** | 0.03 (-0.17 to 0.24) | 0.756 |
| t2-t4 | 0.19 (1.14) | 0.17 | *0.054* | 0.19 (1.12) | 0.17 | **0.050** | -0.01 (-0.27 to 0.26) | 0.957 |
| t1-t4 | -0.24 (1.29) | -0.18 | **0.036** | -0.20 (1.27) | -0.16 | 0.066 | 0.04 (-0.26 to 0.34) | 0.794 |
| Avoidance of physical activity | | | | | | | | |
| t1-t2 | -0.63 (0.95) | -0.66 | **0.000** | -0.81 (0.95) | -0.86 | **0.000** | -0.18 (-0.39 to 0.03) | *0.087* |
| t2-t4 | 0.18 (1.08) | 0.17 | 0.058 | 0.38 (1.06) | 0.36 | **0.000** | 0.20 (-0.05 to 0.46) | 0.116 |
| t1-t4 | -0.50 (1.14) | -0.44 | **0.000** | -0.48 (1.12) | -0.43 | **0.000** | 0.03 (-0.24 to 0.29) | 0.845 |
| Humour/distraction | | | | | | | | |
| t1-t2 | 0.47 (0.78) | 0.60 | **0.000** | 0.58 (0.78) | 0.74 | **0.000** | 0.11 (-0.06 to 0.28) | 0.216 |
| t2-t4 | -0.29 (0.91) | -0.32 | **0.000** | -0.39 (0.90) | -0.44 | **0.000** | -0.10 (-0.32 to 0.11) | 0.352 |
| t1-t4 | 0.22 (0.99) | 0.22 | **0.012** | 0.21 (0.98) | 0.22 | **0.010** | 0 (-0.24 to 0.23) | 0.970 |
| Pain persistence behaviour | | | | | | | | |
| t1-t2 | -0.38 (1.24) | -0.31 | **0.000** | -0.27 (1.22) | -0.22 | **0.006** | 0.11 (-0.16 to 0.38) | 0.430 |
| t2-t4 | -0.18 (1.38) | -0.13 | 0.126 | -0.12 (1.35) | -0.09 | 0.273 | 0.06 (-0.26 to 0.38) | 0.719 |
| t1-t4 | -0.59 (1.44) | -0.41 | **0.000** | -0.40 (1.40) | -0.29 | **0.001** | 0.19 (-0.14 to 0.53) | 0.263 |
| Fear/ Avoidance (TSK) | | | | | | | | |
| t1-t2 | -1.02 (2.74) | -0.37 | **0.000** | -1.67 (2.75) | -0.61 | **0.000** | -0.65 (-1.26 to -0.04) | **0.037** |
| t2-t4 | -0.15 (3.21) | -0.05 | 0.602 | 0.27 (3.15) | 0.08 | 0.317 | 0.41 (-0.35 to 1.17) | 0.286 |
| t1-t4 | -1.11 (3.51) | -0.32 | **0.000** | -1.39 (3.47) | -0.40 | **0.000** | -0.29 (-1.12 to 0.55) | 0.501 |
| Somatic focus (TSK) | | | | | | | | |
| t1-t2 | -1.58 (2.79) | -0.57 | **0.000** | -2.00 (2.76) | -0.72 | **0.000** | -0.42 (-1.04 to 0.21) | 0.190 |
| t2-t4 | -0.06 (3.33) | -0.02 | 0.838 | 0.15 (3.28) | 0.05 | 0.578 | 0.21 (-0.58 to 1.01) | 0.595 |
| t1-t4 | -1.72 (3.70) | -0.47 | **0.000** | -1.77 (3.65) | -0.48 | **0.000** | -0.05 (-0.93 to 0.83) | 0.916 |

BMR=behavioural medical rehabilitation; SET=standard exercise therapy; BET=behavioural exercise therapy; SES=standardised effect size; SD=standard deviation; CI=confidence interval; t1=start of BMR; t2=end of BMR; t3=6 months; t4=12 months; p=significance level; bold=p<0.05; italic=p<0.10; AEQ=Avoidance-Endurance Questionnaire; TSK=Tampa Scale for Kinesiophobia; AEQ=Avoidance-Endurance Questionnaire

### Health-related quality of life (SF-12)

| ***Table* *4*: Health-related quality of life (SF-12)** | | | | | | | | |
| --- | --- | --- | --- | --- | --- | --- | --- | --- |
| **Phase** | **BMR+SET Mean change (SD)** | **SES** | ***p*-value** | **BMR+BET Mean change (SD)** | **SES** | ***p*-value** | **adjusted mean**  **difference (95% CI)** | ***p*-value** |
| Physical Composite Scale | | | | | | | | |
| t1-t2 | 5.54 (7.87) | 0.70 | **0.000** | 5.83 (7.92) | 0.74 | **0.000** | 0.28 (-1.58 to 2.15) | 0.765 |
| t2-t4 | -1.04 (9.35) | -0.11 | 0.212 | -2.78 (8.86) | -0.31 | **0.001** | -1.74 (-3.97 to 0.50) | 0.128 |
| t1-t4 | 4.89 (9.78) | 0.50 | **0.000** | 2.97 (9.31) | 0.32 | **0.000** | -1.91 (-4.25 to 0.43) | 0.111 |
| Mental Composite Scale | | | | | | | | |
| t1-t2 | 8.29 (10.15) | 0.82 | **0.000** | 7.68 (10.25) | 0.75 | **0.000** | -0.62 (-3.03 to 1.80) | 0.617 |
| t2-t4 | -4.27 (12.94) | -0.33 | **0.000** | -1.97 (12.29) | -0.16 | 0.071 | 2.30 (-0.79 to 5.40) | 0.146 |
| t1-t4 | 4.65 (14.64) | 0.32 | **0.000** | 6.17 (14.06) | 0.44 | **0.000** | 1.52 (-1.99 to 5.04) | 0.396 |

BMR=behavioural medical rehabilitation; SET=standard exercise therapy; BET=behavioural exercise therapy; SES=standardised effect size; SD=standard deviation; CI=confidence interval; t1=start of BMR; t2=end of BMR; t3=6 months; t4=12 months; p=significance Level; bold=p<0.05; italic=p<0.10

### Depression (PHQ-D), anxiety (GAD-7), stress (PSS)

| ***Table* 5: Depression (PHQ-D), anxiety (GAD-7), stress (PSS)** | | | | | | | | |
| --- | --- | --- | --- | --- | --- | --- | --- | --- |
| **Phase** | **BMR+SET Mean change (SD)** | **SES** | ***p*-value** | **BMR+BET Mean change (SD)** | **SES** | ***p*-value** | **adjusted mean**  **difference (95% CI)** | ***p*-value** |
| **Depression (PHQ-D)** | | | | | | | | |
| t1-t2 | -4.05 (3.81) | 0.31 | **0.000** | -4.04 (3.85) | 0.31 | **0.000** | 0.01 (-0.84 bis 0.87) | 0.977 |
| t2-t4 | 2.57 (4. 62) | 0.41 | **0.000** | 2.10 (4.53) | 0.38 | **0.000** | -0.47 (-1.56 bis 0.62) | 0.399 |
| t1-t4 | -1.72 (5.17) | 0.45 | **0.000** | -2.29 (5.12) | 0.43 | **0.000** | -0.57 (-1.80 bis 0.65) | 0.360 |
| **Anxiety (GAD-7)** | | | | | | | | |
| t1-t2 | -3.94 (3.72) | 0.30 | **0.000** | -4.39 (3.66) | 0.30 | **0.000** | -0.45 (-1.27 bis 0.38) | 0.289 |
| t2-t4 | 2.35 (4.58) | 0.40 | **0.000** | 2.02 (4.49) | 0.38 | **0.000** | -0.33 (-1.41 bis 0.75) | 0.553 |
| t1-t4 | -1.89 (5.22) | 0.46 | **0.000** | -2.65 (5.16) | 0.43 | **0.000** | -0.76 (-2.00 bis 0.48) | 0.230 |
| **Stress (PSS)** | | | | | | | | |
| t1-t2 | -1.99 (2.90) | 0.23 | **0.000** | -1.8 (2.89) | 0.23 | **0.000** | 0.19 (-0.45 bis 0.83) | 0.565 |
| t2-t4 | 1.33 (3.48) | 0.30 | **0.000** | 0.58 (3.40) | 0.29 | **0.044** | -0.75 (-1.57 bis 0.07) | *0.072* |
| t1-t4 | -0.78 (3.87) | 0.34 | **0.023** | -1.35 (3.79) | 0.32 | **0.000** | -0.57 (-1.48 bis 0.34) | 0.217 |

BMR=behavioural medical rehabilitation; SET=standard exercise therapy; BET=behavioural exercise therapy; SES=standardised effect size; SD=standard deviation; CI=confidence interval; t1=start of BMR; t2=end of BMR; t3=6 months; t4=12 months; p=significance Level; bold=p<0.05; italic=p<0.10; PHQ-D=Patient Health Questionnaire; GAD-7=General Anxiety Disorder 7-item Scale; PSS=Perceived Stress Scale

### Physical activity (FFkA)

| ***Table* *6:* Physical activity** | | | | | | | | |
| --- | --- | --- | --- | --- | --- | --- | --- | --- |
| **Phase** | **BMR+SET Mean change (SD)** | **SES** | ***p*-value** | **BMR+BET Mean change (SD)** | **SES** | ***p*-value** | **adjusted mean**  **difference (95% CI)** | ***p*-value** |
| Total physical activity ^1^ | | | | | | | | |
| t1-t3 | -0.34 (12.44) | -0.03 | 0.753 | 0.31 (12.35) | 0.02 | 0.768 | 0.65 (-2.29 to 3.58) | 0.666 |
| t3-t4 | -0.25 (14.17) | -002 | 0.852 | 0.54 (14.35) | 0.04 | 0.673 | 0.79 (-2.85 to 4.44) | 0.670 |
| t1-t4 | -0.60 (8.58) | -0.07 | 0.470 | 0.84 (8.86) | 0.10 | 0.285 | 1.44 (-0.79 to 3.67) | 0.207 |
| Sport activity^1^ | | | | | | | | |
| t1-t3 | 0.98 (6.37) | 0.15 | 0.077 | 0.91 (6.34) | 0.14 | **0.090** | -0.08 (-1.58 to 1.43) | 0.921 |
| t3-t4 | -0.62 (7.11) | -0.09 | 0.365 | -0.03 (7.20) | 0.00 | 0.968 | 0.59 (-1.23 to 2.41) | 0.526 |
| t1-t4 | 0.37 (3.52) | 0.10 | 0.275 | 0.88 (3.58) | 0.25 | **0.006** | 0.51 (-0.39 to 1.42) | 0.267 |
| Physical activity during leisure time^1^ | | | | | | | | |
| t1-t3 | -0.57 (5.55) | -0.10 | 0.237 | -0.38 (5.52) | -0.07 | 0.418 | 0.2 (-1.11 to 1.51) | 0.769 |
| t3-t4 | 0.16 (6.25) | 0.03 | 0.785 | 0.37 (6.34) | 0.06 | 0.516 | 0.2 (-1.41 to 1.81) | 0.805 |
| t1-t4 | -0.41 (3.57) | -0.11 | 0.233 | -0.01 (3.68) | 0.00 | 0.976 | 0.4 (-0.53 to 1.33) | 0.399 |
| Basic physical activity^1^ | | | | | | | | |
| t1-t3 | -0.67 (6.93) | -0.10 | 0.267 | -0.22 (6.89) | 0.92 | 0.707 | 0.45 (-1.19 to 2.09) | 0.588 |
| t3-t4 | 0.06 (7.92) | 0.01 | 0.933 | 0.19 (8.10) | 1.59 | 0.792 | 0.13 (-1.92 to 2.17) | 0.905 |
| t1-t4 | -0.61 (5.31) | -0.11 | 0.236 | -0.03 (5.59) | 0.94 | 0.954 | 0.58 (-0.81 to 1.97) | 0.416 |

## BMR=behavioural medical rehabilitation; SET=standard exercise therapy; BET=behavioural exercise therapy; SES=standardised effect size; SD=standard deviation; CI=confidence interval; t1=start of BMR; t2=end of BMR; t3=6 months; t4=12 months; p=significance Level; bold=p<0.05; italic=p<0.10

### Determinants of physical activity

| ***Table* *7*: Determinants of physical activity** | | | | | | | | |
| --- | --- | --- | --- | --- | --- | --- | --- | --- |
| **Phase** | **BMR+SET Mean change (SD)** | **SES** | ***p*-value** | **BMR+BET Mean change (SD)** | **SES** | ***p*-value** | **adjusted mean**  **difference (95% CI)** | ***p*-value** |
| Cognitive attitudinal component | | | | | | | | |
| t1-t2 | -0.10 (0.88) | -0.11 | 0.175 | 0.02 (0.87) | 0.02 | 0.766 | 0.12 (-0.08 to 0.31) | 0.240 |
| t2-t4 | 0.04 (0.93) | 0.05 | 0.597 | -0.01 (0.93) | -0.01 | 0.867 | -0.06 (-0.28 to 0.16) | 0.618 |
| t1-t4 | -0.01 (0.84) | -0.02 | 0.860 | 0.02 (0.82) | 0.03 | 0.737 | 0.04 (-0.16 to 0.23) | 0.720 |
| Emotional attitudinal component | | | | | | | | |
| t1-t2 | 0.15 (0.88) | 0.17 | **0.033** | 0.25 (0.87) | 0.29 | **0.001** | 0.10 (-0.10 to 0.29) | 0.332 |
| t2-t4 | -0.18 (0.96) | -0.18 | **0.038** | -0.19 (0.96) | -0.19 | **0.022** | -0.01 (-0.24 to 0.22) | 0.931 |
| t1-t4 | -0.08 (0.89) | -0.09 | 0.321 | -0.04 (0.86) | -0.05 | 0.583 | 0.04 (-0.17 to 0.24) | 0.726 |
| Risk perception | | | | | | | | |
| t1-t2 | -0.12 (0.71) | -0.17 | **0.038** | -0.23 (0.70) | -0.33 | **0.000** | -0.11 (-0.27 to 0.04) | 0.157 |
| t2-t4 | 0.09 (0.79) | 0.12 | 0.187 | 0.16 (0.78) | 0.20 | **0.016** | 0.07 (-0.12 to 0.25) | 0.482 |
| t1-t4 | -0.04 (0.82) | -0.05 | 0.598 | -0.09 (0.80) | -0.11 | 0.191 | -0.05 (-0.24 to 0.14) | 0.609 |
| Self-efficacy | | | | | | | | |
| t1-t2 | 0.12 (0.55) | 0.21 | **0.009** | 0.19 (0.54) | 0.36 | **0.000** | 0.08 (-0.04 to 0.2) | 0.211 |
| t2-t4 | -0.27 (0.63) | -0.43 | **0.000** | -0.4 (0.61) | -0.66 | **0.000** | -0.14 (-0.28 to 0.01) | 0.071 |
| t1-t4 | -0.11 (0.67) | -0.17 | *0.053* | -0.17 (0.65) | -0.26 | **0.003** | -0.05 (-0.21 to 0.10) | 0.503 |
| Positive outcome expectancies | | | | | | | | |
| t1-t2 | -0.02 (0.40) | -0.05 | 0.494 | 0.05 (0.40) | 0.12 | 0.129 | 0.07 (-0.02 to 0.16) | 0.119 |
| t2-t4 | -0.39 (0.48) | -0.82 | **0.000** | -0.46 (0.47) | -0.98 | **0.000** | -0.07 (-0.18 to 0.05) | 0.243 |
| t1-t4 | -0.38 (0.53) | -0.71 | **0.000** | -0.37 (0.52) | -0.72 | **0.000** | 0 (-0.12 to 0.13) | 0.956 |
| Negative outcome expectancies | | | | | | | | |
| t1-t2 | -0.14 (0.44) | -0.33 | **0.000** | -0.13 (0.44) | -0.30 | **0.000** | 0.01 (-0.08 to 0.11) | 0.763 |
| t2-t4 | -0.07 (0.50) | -0.13 | 0.138 | -0.07 (0.50) | -0.15 | 0.084 | -0.01 (-0.13 to 0.11) | 0.906 |
| t1-t4 | -0.21 (0.54) | -0.38 | **0.000** | -0.18 (0.53) | -0.33 | **0.000** | 0.03 (-0.10 to 0.16) | 0.641 |
| Outcome experiences | | | | | | | | |
| t1-t2 | 0.37 (0.7) | 0.56 | **0.000** | 0.47 (0.64) | 0.74 | **0.000** | 0.11 (-0.04 to 0.25) | 0.763 |
| t2-t4 | -0.45 (0.72) | -0.62 | **0.000** | -0.51 (0.70) | -0.73 | **0.000** | -0.06 (-0.23 to 0.11) | 0.466 |
| t1-t4 | -0.02 (0.74) | -0.02 | 0.797 | 0.03 (0.72) | 0.04 | 0.604 | 0.05 (-0.13 to 0.22) | 0.587 |
| Intention | | | | | | | | |
| t1-t2 | 0.16 (0.51) | 0.31 | **0.000** | 0.27 (0.51) | 0.52 | **0.000** | 0.11 (0 to 0.22) | *0.058* |
| t2-t4 | -0.42 (0.61) | -0.70 | **0.000** | -0.54 (0.59) | -0.90 | **0.000** | -0.11 (-0.25 to 0.03) | 0.131 |
| t1-t4 | -0.22 (0.66) | -0.33 | **0.000** | -0.21 (0.65) | -0.33 | **0.000** | 0.01 (-0.15 to 0.16) | 0.914 |
| Action planning | | | | | | | | |
| t1-t2 | 0.34 (0.64) | 0.53 | **0.000** | 0.52 (0.63) | 0.83 | **0.000** | 0.19 (0.05 to 0.33) | **0.009** |
| t2-t4 | -0.23 (0.75) | -0.30 | **0.001** | -0.36 (0.74) | -0.49 | **0.000** | -0.13 (-0.31 to 0.05) | 0.150 |
| t1-t4 | 0.14 (0.83) | 0.17 | *0.054* | 0.2 (0.82) | 0.25 | **0.004** | 0.06 (-0.14 to 0.25) | 0.554 |
| Action control | | | | | | | | |
| t1-t2 | 0.85 (0.74) | 1.16 | **0.000** | 0.86 (0.73) | 1.17 | **0.000** | 0 (-0.16 to 0.17) | 0.969 |
| t2-t4 | -0.70 (0.82) | -0.86 | **0.000** | -0.63 (0.80) | -0.79 | **0.000** | 0.07 (-0.12 to 0.27) | 0.451 |
| t1-t4 | 0.25 (0.84) | 0.30 | **0.001** | 0.28 (0.82) | 0.34 | **0.000** | 0.03 (-0.17 to 0.23) | 0.783 |

BMR=behavioural medical rehabilitation; SET=standard exercise therapy; BET=behavioural exercise therapy; SES=standardised effect size; SD=standard deviation; CI=confidence interval; t1=start of BMR; t2=end of BMR; t3=6 months; t4=12 months; p=significance level; bold=p<0.05; italic=p<0.10

Table 8: Unadjusted Means (SD) for each Study Group

| **Outcome** | **Baseline** | | | | | **4 weeks** | | | | | **6 months** | | | | | **12 months** | | | |
| --- | --- | --- | --- | --- | --- | --- | --- | --- | --- | --- | --- | --- | --- | --- | --- | --- | --- | --- | --- |
|  | **BMR+**  **BET** |  | **BMR+**  **SET** |  | **BMR+**  **BET** | |  | **BMR+**  **SET** |  | **BMR+**  **BET** | |  | **BMR+**  **SET** |  | **BMR+BET** | |  | **BMR+**  **SET** |  |
|  | **Mean (SD)** | **N** | **Mean (SD)** | **N** | **Mean (SD)** | | **N** | **Mean (SD)** | **N** | **Mean (SD)** | | **N** | **Mean (SD)** | **N** | **Mean (SD)** | | **N** | **Mean (SD)** | **N** |
| **Primary Outcome** | | | | | | | | | | | | | | | | | | | |
| Functional  Ability | 63.33 (19.95) | 164 | 62.28 (20.50) | 163 | 69.27 (20.76) | | 157 | 68.14 (21.10) | 157 | 68.55 (24.37) | | 142 | 68.32 (21.41) | 132 | 67.83 (23.19) | | 129 | 68.50 (22.55) | 110 |
| **Secondary Outcome** | | | | | | | | | | | | | | | | | | | |
| Pain (NRS) | 59.99 (16.06) | 162 | 59.99 (17.44) | 159 | 54.07 (16.78) | | 156 | 54.61 (15.26) | 157 | 49.73 (20.65) | | 132 | 49.38 (20.01) | 141 | 45.40 (20.46) | | 126 | 44.16 (21.66) | 109 |
| Physical Composite Scale (SF-12) | 36.23 (8.39) | 152 | 35.46 (9.03) | 152 | 42.06 (9.14) | | 140 | 41.01 (9.39) | 135 | 40.67 (10.25) | | 129 | 40.49 (9.60) | 127 | 39.28 (10.14) | | 126 | 39.97 (9.98) | 107 |
| Mental Composite Scale (SF-12) | 39.97 (11.92) | 152 | 40.32 (12.01) | 152 | 47.64 (11.59) | | 140 | 48.61 (9.93) | 135 | 46.66 (12.07) | | 129 | 46.47 (10.59) | 127 | 45.67 (11.74) | | 126 | 44.33 (11.90) | 107 |
| Depression (PHQ-D) | 10.01 (5.16) | 157 | 9.65 (4.66) | 160 | 5.97 (4.07) | | 155 | 6.89 (4.66) | 153 | 7.02 (5.33) | | 140 | 6.89 (4.66) | 130 | 8.07 (4.87) | | 127 | 8.18 (5.09) | 109 |
| Anxiety (GAD-7) | 9.29 (5.02) | 163 | 8.64 (4.83) | 159 | 4.90 (3.71) | | 154 | 4.69 (3.75) | 153 | 5.90 (4.56) | | 141 | 5.87 (3.78) | 129 | 6.91 (4.29) | | 128 | 7.04 (4.68) | 107 |
| Stress (PSS) | 7.83 (3.39) | 162 | 7.78 (3.20) | 160 | 6.03 (3.42) | | 156 | 5.79 (3.18) | 155 | 6.32 (3.62) | | 141 | 6.46 (3.37) | 132 | 6.61 (3.46) | | 128 | 7.12 (3.46) | 109 |
| Action-oriented Coping (FESV) | 15.02 (4.75) | 153 | 14.80 (4.81) | 162 | 18.21 (4.03) | | 149 | 17.40 (4.28) | 147 | 17.55 (4.61) | | 140 | 16.87 (4.08) | 132 | 16.88 (4.22) | | 127 | 16.34 (4.44) | 110 |
| Cognitive Restructuring (FESV) | 13.47 (4.47) | 153 | 13.03 (4.58) | 162 | 17.04 (3.94) | | 148 | 16.17 (4.59) | 147 | 15.93 (4.60) | | 140 | 15.53 (4.23) | 132 | 14.82 (4.20) | | 127 | 14.89 (4.66) | 110 |
| Subjective Coping Competence (FESV) | 15.21 (4.41) | 153 | 15.45 (4.63) | 161 | 17.50 (3.96) | | 148 | 17.19 (4.22) | 147 | 16.88 (4.53) | | 141 | 16.65 (4.14) | 132 | 16.26 (4.04) | | 129 | 16.11 (3.95) | 110 |
| Mental Distraction (FESV) | 10.39 (4.94) | 153 | 10.58 (4.67) | 161 | 12.80 (4.69) | | 148 | 12.84 (4.69) | 147 | 11.70 (4.91) | | 141 | 11.65 (4.25) | 132 | 10.61 (4.36) | | 128 | 10.45 (4.82) | 110 |

| *Table 8*: Unadjusted Means (SD) for each Study Group (continued) | | | | | | | | | | | | | | | | |
| --- | --- | --- | --- | --- | --- | --- | --- | --- | --- | --- | --- | --- | --- | --- | --- | --- |
| Counter Activities (FESV) | 12.39 (4.44) | 153 | 13.21 (4.37) | 161 | 14.10 (4.28) | 148 | 14.02 (4.23) | 147 | 13.47 (4.78) | 141 | 13.36 (4.09) | 132 | 12.84 (4.18) | 128 | 12.70 (4.32) | 110 |
| Relaxation (FESV) | 11.17 (4.66) | 153 | 10.77 (4.92) | 161 | 14.59 (4.53) | 148 | 14.65 (4.82) | 147 | 13.64 (5.37) | 141 | 13.56 (4.78) | 132 | 12.70 (4.78) | 128 | 12.48 (5.09) | 110 |
| Catastrophizing (AEQ) | 0.92 (1.15) | 164 | 0.77 (1.11) | 161 | 0.57 (0.92) | 156 | 0.59 (1.01) | 157 | 0.68 (1.07) | 142 | 0.61 (0.88) | 132 | 0.80 (0.99) | 129 | 0.62 (0.91) | 110 |
| Help-Hopelessness (AEQ) | 2.52 (1.22) | 164 | 2.45 (1.21) | 161 | 1.77 (1.32) | 156 | 1.86 (1.23) | 157 | 1.95 (1.33) | 142 | 1.90 (1.22) | 132 | 2.13 (1.36) | 129 | 1.93 (1.31) | 110 |
| Thought Suppression (AEQ) | 3.72 (0.94) | 163 | 3.64 (0.90) | 163 | 3.75 (0.99) | 156 | 3.66 (0.94) | 156 | 3.60 (0.96) | 142 | 3.55 (1.02) | 132 | 3.44 (0.95) | 128 | 3.45 (0.96) | 110 |
| Anxiety/ Depression (AEQ) | 2.77 (1.28) | 164 | 2.64 (1.16) | 161 | 2.00 (1.32) | 157 | 1.77 (1.33) | 156 | 2.25 (1.32) | 141 | 2.05 (1.24) | 131 | 2.50 (1.29) | 129 | 2.33 (1.31) | 110 |
| Positive Mood (AEQ) | 3.18 (1.41) | 164 | 3.22 (1.24) | 159 | 3.91 (1.29) | 157 | 3.89 (1.20) | 156 | 3.63 (1.37) | 141 | 3.68 (1.22) | 131 | 3.36 (1.33) | 129 | 3.47 (1.26) | 110 |
| Avoidance of Social Activities (AEQ) | 2.07 (1.33) | 163 | 1.86 (1.26) | 162 | 1.72 (1.30) | 156 | 1.47 (1.22) | 155 | 1.81 (1.25) | 142 | 1.57 (1.25) | 132 | 1.90 (1.26) | 128 | 1.66 (1.39) | 110 |
| Avoidance of Physical Activities (AEQ) | 3.49 (0.98) | 163 | 3.31 (1.02) | 163 | 2.67 (1.11) | 156 | 2.68 1.01 | 156 | 2.86 (0.97) | 142 | 2.77 1.04 | 132 | 3.05 (0.94) | 128 | 2.86 (1.12) | 110 |
| Humour/ Distraction (AEQ) | 3.01 (1.04) | 163 | 3.07 (0.89) | 162 | 3.59 (1.02) | 156 | 3.54 (0.95) | 155 | 3.39 (1.08) | 142 | 3.39 (0.94) | 132 | 3.20 (0.98) | 128 | 3.25 (0.89) | 110 |
| Pain Persistence Behavior (AEQ) | 3.62 (1.34) | 164 | 3.68 (1.24) | 160 | 3.35 (1.53) | 156 | 3.30 (1.39) | 157 | 3.29 (1.42) | 142 | 3.21 (1.37) | 132 | 3.23 (1.50) | 129 | 3.12 (1.44) | 110 |
| Fear/ Avoidance (TSK) | 7.54 (3.53) | 158 | 6.87 (3.26) | 158 | 5.86 (3.24) | 157 | 5.85 (2.89) | 153 | 6.00 (3.26) | 141 | 5.78 (3.28) | 129 | 6.13 (3.49) | 127 | 5.71 (3.38) | 108 |
| Somatic Focus (TSK | 7.17 (3.45) | 160 | 6.78 (3.43) | 159 | 5.17 (3.15) | 151 | 5.20 (3.21) | 152 | 5.25 (3.45) | 140 | 5.17 (3.12) | 128 | 5.33 (3.55) | 127 | 5.14 (3.29) | 106 |
| Basic Physical Activity (FFkA) | 2.44 (4.80) | 163 | 2.89 (6.65) | 161 | - | - | - | - | 2.32 (3.99) | 142 | 2.25 (2.60) | 132 | 2.41 (3.18) | 128 | 2.28 (2.59) | 109 |
| Physical Activity during Leisure Time (FFkA) | 2.72 (3.27) | 164 | 3.22 (4.39) | 161 | - | - | - | - | 2.53 (2.48) | 142 | 2.73 (2.96) | 132 | 2.71 (3.14) | 127 | 2.81 (2.98) | 109 |

| Table 8: Unadjusted Means (SD) for each Study Group (continued) | | | | | | | | | | | | | | | | |
| --- | --- | --- | --- | --- | --- | --- | --- | --- | --- | --- | --- | --- | --- | --- | --- | --- |
| Sport Activity (FFkA) | 1.58 (2.64) | 164 | 1.69 (2.74) | 162 | - | - | - | - | 2.48 (4.14) | 142 | 2.37 (3.09) | 132 | 2.47 (3.45) | 128 | 2.06 (3.00) | 110 |
| Total Physical Activity (FFkA) | 6.73 (7.47) | 163 | 7.82 (10.63) | 161 | - | - | - | - | 7.30 (6.92) | 142 | 7.35 (6.26) | 132 | 7.57 (7.65) | 127 | 7.22 (6.83) | 109 |
| Cognitive Attitudinal Component | 5.28 (0.80) | 164 | 5.32 (0.77) | 161 | 5.30 (0.70) | 155 | 5.22 (0.73) | 157 | 5.29 (0.90) | 142 | 5.24 (0.74) | 131 | 5.29 (0.68) | 129 | 5.26 (0.71) | 110 |
| Emotional Attitudinal Component | 4.70 (0.88) | 164 | 4.75 (0.81) | 160 | 4.95 (0.68) | 155 | 4.90 (0.76) | 157 | 4.86 (0.87) | 142 | 4.81 (0.89) | 132 | 4.77 (0.81) | 128 | 4.72 (0.88) | 110 |
| Intention | 3.29 (0.50) | 164 | 3.30 (0.54) | 162 | 3.56 (0.41) | 156 | 3.46 (0.43) | 155 | 3.29 (0.61) | 142 | 3.25 (0.57) | 132 | 3.03 (0.62) | 129 | 3.04 (0.53) | 109 |
| Self-Efficacy | 3.19 (0.60) | 164 | 3.20 (0.55) | 162 | 3.39 (0.54) | 156 | 3.32 (0.51) | 155 | 3.18 (0.60) | 141 | 3.18 (0.59) | 132 | 2.98 (0.62) | 129 | 3.05 (0.60) | 109 |
| Action Planning | 2.85 (0.81) | 162 | 2.88 (0.74) | 162 | 3.37 (0.53) | 155 | 3.21 (0.50) | 157 | 3.19 (0.71) | 141 | 3.10 (0.70) | 132 | 3.02 (0.69) | 129 | 2.99 (0.75) | 109 |
| Risk Perception | 3.28 (0.76) | 162 | 3.23 (0.73) | 161 | 3.05 (0.75) | 154 | 3.11 (0.71) | 157 | 3.13 (0.86) | 142 | 3.16 (0.78) | 131 | 3.21 (0.81) | 129 | 3.20 (0.84) | 108 |
| Positive Outcome Expectancies | 3.45 (0.42) | 164 | 3.41 (0.45) | 163 | 3.50 (0.45) | 157 | 3.39 (0.45) | 157 | 3.26 (0.55) | 142 | 3.19 (0.50) | 131 | 3.03 (0.51) | 129 | 3.00 (0.51) | 109 |
| Negative Outcome Expectancies | 1.90 (0.54) | 164 | 1.93 (0.53) | 162 | 1.78 (0.53) | 155 | 1.78 (0.50) | 157 | 1.74 (0.46) | 142 | 1.75 (0.50) | 131 | 1.70 (0.47) | 129 | 1.72 (0.50) | 109 |
| Outcome Experiences | 2.74 (0.80) | 164 | 2.73 (0.65) | 160 | 3.21 (0.60) | 157 | 3.10 (0.63) | 156 | 2.95 (0.71) | 141 | 2.87 (0.72) | 132 | 2.70 (0.73) | 129 | 2.65 (0.70) | 110 |
| Action Control | 2.54 (0.78) | 162 | 2.49 (0.67) | 160 | 3.40 (0.52) | 157 | 3.34 (0.55) | 155 | 3.08 (0.67) | 141 | 2.99 (0.67) | 132 | 2.77 (0.69) | 129 | 2.64 (0.73) | 109 |

BMR=behavioural medical rehabilitation, SET=standardized exercise therapy, BET=behavioural exercise therapy, SD=standard deviation, N=number of participants, SD=standard deviation, HFAQ=Hannover Functional Ability Questionnaire, NRS=Numeric Rating Scale, SF-12=Short-Form-12, PHQ-D=Patient Health Questionnaire, GAD-7=General Anxiety Disorder 7-item Scale, PSS=Perceived Stress Scale, FESV=Pain Management Questionnaire, AEQ=Avoidance-Endurance Questionnaire, TSK=Tampa Scale for Kinesiophobia, FFkA=Freiburg Questionnaire on Physical Activity.
